# Finding Functional Differences Between Species in a Microbial Community: Case Studies in Wine Fermentation and Kefir Culture

**DOI:** 10.3389/fmicb.2019.01347

**Published:** 2019-06-25

**Authors:** Chrats Melkonian, Willi Gottstein, Sonja Blasche, Yongkyu Kim, Martin Abel-Kistrup, Hentie Swiegers, Sofie Saerens, Nathalia Edwards, Kiran R. Patil, Bas Teusink, Douwe Molenaar

**Affiliations:** ^1^Systems Bioinformatics, VU University Amsterdam, Amsterdam, Netherlands; ^2^European Molecular Biology Laboratory, Heidelberg, Germany; ^3^Christian Hansen A/S, Hørsholm, Denmark

**Keywords:** microbial communities, lactic acid bacteria, genomes, metagenomics, computational biology, wine, kefir

## Abstract

Microbial life usually takes place in a community where individuals interact, by competition for nutrients, cross-feeding, inhibition by end-products, but also by their spatial distribution. Lactic acid bacteria are prominent members of microbial communities responsible for food fermentations. Their niche in a community depends on their own properties as well as those of the other species. Here, we apply a computational approach, which uses only genomic and metagenomic information and functional annotation of genes, to find properties that distinguish a species from others in the community, as well as to follow individual species in a community. We analyzed isolated and sequenced strains from a kefir community, and metagenomes from wine fermentations. We demonstrate how the distinguishing properties of an organism lead to experimentally testable hypotheses concerning the niche and the interactions with other species. We observe, for example, that *L. kefiranofaciens*, a dominant organism in kefir, stands out among the *Lactobacilli* because it potentially has more amino acid auxotrophies. Using metagenomic analysis of industrial wine fermentations we investigate the role of an inoculated *L. plantarum* in malolactic fermentation. We observed that *L. plantarum* thrives better on white than on red wine fermentations and has the largest number of phosphotransferase system among the bacteria observed in the wine communities. Also, *L. plantarum* together with *Pantoea, Erwinia, Asaia, Gluconobacter*, and *Komagataeibacter* genera had the highest number of genes involved in biosynthesis of amino acids.

## 1. Introduction

Lactic acid bacteria (LAB) are a group of microorganisms widely used for production of fermented food. They play a key role as natural fermentors or are used as starting cultures for a large variety of foods (Teusink and Molenaar, 2017), such as dairy products, kefir and yogurt (Prado et al., 2015). LAB are also used in alcoholic beverage production with a prominent role in winemaking, due to their capacity to perform malolactic fermentation (MLF) (Lonvaud-Funel, 1999, 2002). In none of these environments do they live in isolation but rather in communities of microscopic and macroscopic scale, for example on the skin and in biofilms. Therefore, LAB should be studied not only in isolation but also as a part of communities. Consequently, there is a strong desire to understand their roles in microbial communities, for example in their stability of communities. A deep understanding of these roles would enable alterations or even design of communities that serve a certain purpose. Results in this direction have already been achieved for small consortia, usually consisting of two species (Song et al., 2014; Biggs et al., 2015; Zomorrodi and Segrè, 2016). However, interactions in natural communities consisting of dozens to thousands of species are hard to analyze.

For complex communities, dynamic abundance data has been used to infer interactions between species within a community (Faust and Raes, 2012). While this can indeed lead to testable predictions, these results can also be very hard to interpret as they do not provide any detail of their underlying mechanism. For example, a positive correlation between two species can be caused by niche-overlap, cross-feeding or because these two species are both affected by a third one (Faust and Raes, 2012). To distinguish these options, the metabolic potential of the individual species should be taken into account as many of the interactions will probably take place at the level of exchange of metabolic products. These analyses currently typically require large-scale metabolic models (Freilich et al., 2010, 2011; Harcombe et al., 2014; Zomorrodi and Segrè, 2016). The reconstruction of such models is a time-consuming process as it usually requires manual curation, experimental validation, gap-filling, and an organism-specific biomass composition. As typically only a small percentage of species within a community can be cultured individually, the generation of high quality models for all members of a community is close to infeasible.

Attempts to do so (Magnusdottir et al., 2017) suffer from a lack of detailed validation of the predictions. Therefore, approaches that rely on genome-scale stoichiometric models are currently mostly applicable to small well-described (synthetic) communities (Mahadevan and Henson, 2012; Harcombe et al., 2014; Song et al., 2014; Biggs et al., 2015; Tan et al., 2015; Zomorrodi and Segrè, 2016) but even there one encounters many technical and biological challenges (Gottstein et al., 2016).

In this paper, we use a purely data-driven approach, with genomic information as primary input, that allows the creation of hypotheses about metabolic and other physiological properties of species in communities without the need to reconstruct detailed genome-scale metabolic models. The starting point of this analysis is gene annotation; we use the KEGG Orthology (KO) Database (Kanehisa and Goto, 2000; Kanehisa et al., 2012) whereby each KO represents a group of gene orthologs from different organisms associated with a molecular function. As KO's alone can be hard to interpret, we also map these KO's on KEGG pathways. This higher level mapping reveals discriminating features between organisms and leads to testable hypotheses about their metabolic and physiological characteristics. Although we used the KEGG annotation tool and database, alternative resources such as Gene ontology(GO), SEED and MetaCyc (Ashburner et al., 2000; Overbeek et al., 2005; Caspi et al., 2016) could be used and yield comparable results (Mitra et al., 2011; Altman et al., 2013).

We apply this computational pipeline on two different case studies. Firstly, to investigate Kefir a fermented milk product made with kefir grains, which consist of a complex microbial community embedded in a polysaccharide matrix. These communities consist of dozens of species (Walsh et al., 2016) whose metabolic capacities are largely elusive. Studies of the kefir community using metagenomic barcoding already showed that Lactobacillus was the most abundant genus, specifically the species *Lactobacillus kefiranofaciens, Lactobacillus buchneri* and *Lactobacillus helveticus* (Nalbantoglu et al., 2014). We expect that knowledge of their metabolism will provide more insight in their interactions in kefir and, therefore, we investigated genomes of 30 organisms isolated from kefir for their potential metabolism.

The second application of the pipeline is in understanding the role of *L. Plantarum* MW-1 in winemaking, by a functional comparison of microbial communities in three varieties of wine. Microbial activities are crucial in the formation of wine flavor and aroma. A prerequisite for improving winemaking is to understand the dynamics of the microbial communities in wine and the interactions that take place during the fermentation (Tempère et al., 2018). The alcoholic fermentation (AF) at the initial stage of winemaking is performed mainly by *Saccharomyces cerevisiae*. Subsequently, *Oenococcus oeni*, which due to its overall resistance to the harsh conditions of wine fermentation, such as high alcohol concentrations, is the best candidate to start a MLF (Ribéreau-Gayon et al., 2006a,b). Various studies indicate the possibility to use alternative MLF starters. *L. plantarum* strains received interest to fulfill this role (Hernandez et al., 2007; Testa et al., 2014), due to their characteristic fermentation profile. To investigate the influence of *L. Plantarum* MW-1 on the development of the microbial communities we followed its inoculation in three different wine varieties (Bobal, Tempranillo, and Airen) from La Mancha, Spain, 2013 (one inoculated and two control fermentations per variety Figures S5, S6). The point of inoculation was chosen to be at the start, to give precedence of MLF over AF. In this way, a reduction of total fermentation time is obtainable, and inhibition of *L. plantarum* by high alcohol levels is avoided. We used metagenome shotgun time-series from these fermentations to study the community. Although next-generation sequencing (NGS) has recently been applied in food research and particular in wine fermentation (Kioroglou et al., 2018; Stefanini and Cavalieri, 2018), the usage of metagenomic shotgun sequencing that allows a direct identification and comparison of the functional potential capabilities for a microbial community and its members, is not yet fully exploited (Morgan et al., 2017a; Sternes et al., 2017; Zepeda-Mendoza et al., 2018).

## 2. Materials and Methods

### 2.1. DNA Extraction and Genome Sequencing of Kefir Isolates

Two milliliters of the culture were pelleted at 15,000 rpm in a table centrifuge. The pellet was suspended in 600 μl TES buffer (25mM Tris; 10mM EDTA; 50mM sucrose) containing 20 mg/ml lysozyme (Sigma-Aldrich, cat# 62971) and incubated for 30 min at 37°C. The samples were then crushed with 0.3 g glass beads (Sigma-Aldrich, cat# G1277, 212–300 μm) at 4m/s for five times 20 s using the FastPrep-24 instrument (MP Biomedicals). 150 μl 20% SDS was added and after 5 min incubation at room temperature the tubes were centrifuged at maximum speed for 2 min. The supernatant was digested with 10 μl proteinase K (20 mg/ml) for 30 min at 37°C and proteins were precipitated with 200 μl potassium acetate (5 M) for 15 min on ice. The samples were then centrifuged for 15 min at 4°C and the supernatant applied to phenol/chloroform extraction. DNA was precipitated by adding two volumes of ice-cold isopropanol and 20 min incubation at –20°C followed by washing with 70% ethanol at 4°C. DNA quality was checked on agarose gel.

Kefir species were identified by Sanger sequencing of the 16S/ITS (internal transcribed spacer) region, using the primers S-D-Bact-0515-a-S-16 (GTGCCAGCMGCNGCGG) and S-*-Univ-1392-a-A-15 (ACGGGCGGTGTGTRC) (Klindworth et al., 2012). Unique isolates were sequenced using the Illumina HiSeq 2000 platform at EMBL genomics core facility (Heidelberg, Germany) with 100 bp paried-end reads. The A5-miseq pipeline was used for quality-based trimming and filtering, error correction and de novo assembly (Coil et al., 2015). The assembled genome was annotated using Prokka version 1.11 (Seemann, 2014).

### 2.2. Sampling and Sequencing of Wine Fermentations

Wine was sampled in the autumn of 2013 at Bodegas Purisima Concepcion (La Mancha, Spain) before the fermentation (day 0), during fermentation (days 1,2,3,4,7,14) and at the end of the fermentation (day 21). Samples of the white wine were taken from the top of the concrete tank by rapidly lowering a 250 mL baby bottle (single use) to 1 m depth using a rope and slowly bring it to the top. The wine was decanted to a 50 mL falcon tube and put directly in a –50°C freezer. To avoid the grape skin cap the red wine was sampled from the valve in the bottom after flushing the valve in order avoid obtaining residue wine. This was also done after racking of the wine. Cautions where taken in order to minimize contamination. Samples were handled wearing gloves and changed between replicates, aluminum foil was applied on the work station and changed between replicates, and filter pipettes were used all the time.

For DNA isolation, cells were pelleted from 50 mL of wine centrifuged at 4,500 g for 10 minutes and subsequently washed three times with 10 mL of 4°C phosphate buffered saline (PBS). The pellet was mixed with G2-DNA enhancer (Ampliqon, Odense, Denmark) in 2 ml tubes and incubated at RT for 5 min. Subsequently, 1 mL of lysis buffer (20 mM Tris-HCl- pH 8.0, 2 mM EDTA and 40 mg/ml lysozyme) was added to the tube and incubated at 37°C for 1 h. An additional 1 mL of CTAB/PVP lysis buffer (50) was added to the lysate and incubated at 65°C for 1 h. DNA was purified from 1 mL of lysate with an equal volume of phenol-chloroform-isoamyl alcohol mixture 49.5:49.5:1 and the upper aqueous layer was further purified with a MinElute PCR Purification kit and the QIAvac 24 plus (Qiagen, Hilden, Germany), according to manufacturer's instructions, and finally eluted in 100 ul DNase-free water.

Prior to library building, genomic DNA was fragmented to an average length of 400 bp using the Bioruptor XL (Diagenode, Inc.), with the profile of 20 cycles of 15 s of sonication and 90 s of rest. Sheared DNA was converted to Illumina compatible libraries using NEBNext library kit E6070L (New England Biolabs) and blunt-ended library adapters described by Meyer and Kircher (2010). The libraries were amplified in 25-mL reactions, with each reaction containing 5 muL of template DNA, 2,5 U AccuPrime Pfx Supermix (Invitrogen, Carlsbad, CA), 1X Accuprime Pfx Supermix, 0.2 uM IS4 forward primer and 0.2 uM reverse primer with sample specific 6 bp index. The PCR conditions were 2 minutes at 95°C to denature DNA and activate the polymerase, 11 cycles of 95°C for 15 s, 60°C annealing for 30 s, and 68°C extension for 40 s, and a final extension of 68°C extension for 7 minutes.

The quality and quantity of the libraries were measured using the high sensitivity DNA analysis kit on the Bioanalyzer 2100 (Agilent technologies, Santa Clara, United States), and the libraries were pooled at equimolar concentration. Sequencing was performed on the Illumina HiSeq 2500 in PE100 mode and MiSeq in 250PE mode following the manufacturer's instructions.

### 2.3. General Workflow of Functional Computational Analysis

**Figure 1:**
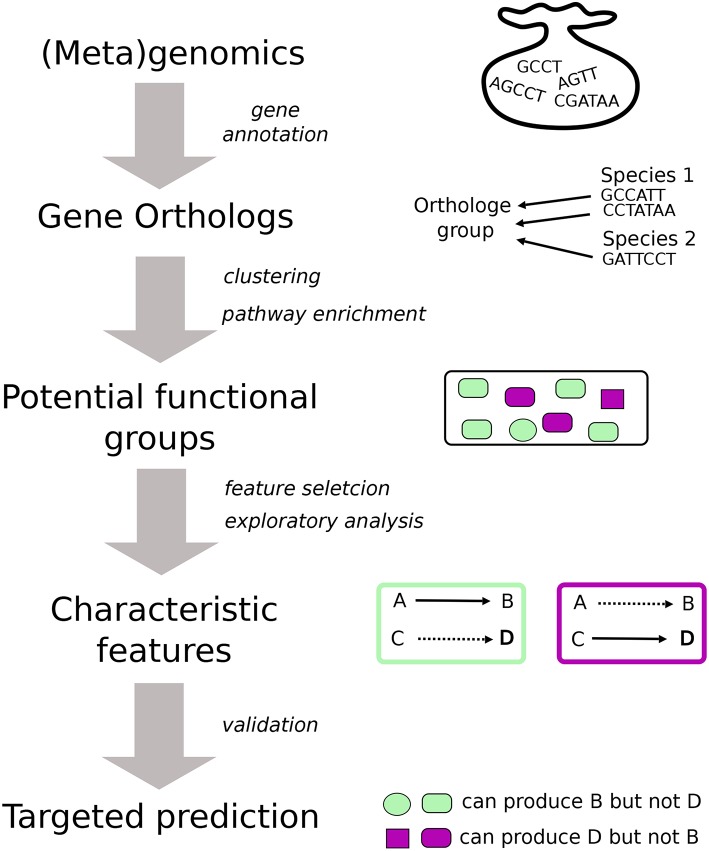
Graphical representation of the general work-flow for the functional analysis. Starting point of the analysis is a gene annotation which is carried out using BlastKoala and GhostKoala in this study. We use the gene orthologs (KO's) to cluster species and samples based on their KO content. From the individual clusters we extract the characteristic features which leads to educated predictions about the functional potential of individual species and a community present in a sample. In the case of isolates, the predictions are confirmed using MetaDraft that does not rely on KO's.

The general workflow that we follow is illustrated in Figure 1. The starting point of the analysis is gene annotation to determine orthologous genes (Gabaldn and Koonin, 2013) for which we use BlastKoala and GhostKoala (Kanehisa et al., 2016), through webservices provided by KEGG (Kanehisa et al., 2013). These webservices map genes to KEGG Orthologs (KO's) that represent groups of orthologous genes which are linked to a molecular-level function. Based on their KO content, the organisms and samples can be clustered. This process yields several groups of distinct characteristics that are determined using diverse data mining techniques and mainly, but not exclusively, concern the metabolic potential. Finally, these characteristics enable the formulation of specific hypotheses about the physiological properties of species and a community as a whole in individual samples. Other methods for the analysis of genome information on the functional level exist, such as MG-RAST (Meyer et al., 2008) and Megan (Quince et al., 2017). HUMAnN (Abubucker et al., 2012) was the first to incorporate microbial pathway abundances for metagenomic data. We choose to apply a custom pipeline to be generic and allow high versatilely throughout the analysis. Moreover, the use of the published BlastKOALA and GhostKOALA from KEGG (Kanehisa et al., 2016) provides an up to date annotation with KEGG database. Alternative, eggNOG (Huerta-Cepas et al., 2016) provide a strong framework for orthology annotation. All figures were visualized using base R packages (R Core Team, 2016), ggplot2 (Wickham, 2016) and pheatmap (Raivo, 2019).

### 2.4. Metagenomic Sequence Prepossessing

Quality control and filtering was applied on all paired-read data using FastQC v0.11.4 (Andrews, 2010) before and after the application of Trim Galore v0.4.1 (Andrews, 2012) and Cutadapt v1.9.1 (Martin, 2011), tools for quality and adapter trimming. Subsequently, the reconstruction of full-length small subunit (SSU rRNA) gene sequences was obtained using EMIRGE (Miller et al., 2011) with the SILVA 123 SSURef Nr99 database (Pruesse et al., 2007). A taxonomy was assigned using SINA Alignment Service on the resulting SSUs (Pruesse et al., 2012). The resulting SSU's were clustered to OTUs with 97% identity using UCLUST (Edgar, 2010) and the estimates of relative taxon abundances provided by the program added and normalized accordingly. A chimera sequence check was performed using UCHIME (Edgar, 2016). For both tools the qiime interface was used (Caporaso et al., 2010). Afterwards, the OTUs were arranged to a BIOM table with a custom R script (R Core Team, 2016), to allow further analysis.

### 2.5. Sequence Binning

For each grape variety the metagenome shotgun samples were merged together to achieve deep coverage, and were assembled with the Iterative De Bruijn graph de novo Assembler for short reads sequencing data with highly Uneven sequencing Depth (IDBA-UD) (Peng et al., 2012). The resulting contigs were binned with Maxbin 2.0 (Wu et al., 2014, 2015), which clusters the sequences into draft genomes (bins) using the tetranucleotide frequencies and sequence coverage. For differential coverage, all the metagenome samples belong to fermantaitons of the same grape variety were used. Furthermore, bin taxonomy assignments were carried out following the multi-metagenome pipeline (Albertsen et al., 2013). Maxbin calculates a quality of the resulting bins, using occurrence of essential genes to calculate a completeness score for the entire bin.

### 2.6. Gene Annotation

The gene annotation was carried out using BlastKoala and GhostKoala (Kanehisa et al., 2016) using the databases, “genus_prokaryotes” and “genus_prokaryotes” or “genus_prokaryotes plus family_eukaryotes” for the kefir isolates and the metagenomic samples, respectively. While protein fasta files can be directly submitted to BlastKoala when isolates are examined, a re-assembly with IDBA-UD was necessary before submission of metagenome samples (Peng et al., 2012). To predict the open reading frames (ORFs), we used prodigal (Hyatt et al., 2012) with parameterization for metagenome data. The produced ORFs are then used as an input for GhostKOALA, which provides the KO (KEGG Orthology) assignments. Also, the effect of different sequencing depth on the number of predicted ORFs was investigated Figure S7.

### 2.7. Calculation of Feature Matrices and Clustering

Using the output from BlastKoala and GhostKoala, several feature matrices were calculated. In the case of microbial isolates, a feature matrix *K* is constructed of dimensions *n* × *m* where *m* is the number of isolated species and *n* is the number of KO's. The entries *k*_*ij*_ are 1 if the KO *j* is present in species *i* and 0 otherwise. A *r* × *m* feature matrix *P* was calculated, whose *r* rows and *m* columns correspond to KEGG pathway ID's and isolated species, respectively. The entries *p*_*ij*_ thereby represent the number of KO's present in pathway *i* for species *j*. To account for the different pathways sizes, *p*_*ij*_ is normalized with respect to the total number of KO's present in pathway *i*.

For the analysis of the metagenomic data, a *n* × *m* feature matrix *G* was constructed by calculating sequence abundance per KO and summing these per genus. The entries *g*_*ij*_ equal the number of sequence reads of the genus *i* present in sample *j*. To account for variability in sequence reads per sample the entries *g*_*ij*_ were normalized with respect to the number of sequence reads per sample *g*_*j*_ and multiplied by 1 million ($\frac{g_{ij}}{g_{j}}\times 10^{6}$). We also took into account the inoculation of *Lactobacillus plantarum* and further normalize all samples using the complement (1 − *g*_*lactobacillus*_) of the *Lactobacillus* genus abundance ($\frac{g_{ij}}{1-g_{lactobacillus}}$)

Another feature matrix *A* was calculated in which entries *a*_*ij*_ equal the number of sequence reads mapped to a KO-genus combination *i* present in sample *j*. This matrix yields a very large number of features and, consequently, very detailed information.

Finally, a feature matrix *PM* is used to explore biological implications by mapping KO's to KEGG pathways. Similarly, *m* is the samples during the fermentations, on the other hand *n* now is the KEGG pathway IDs tagged with genera. The entries *pm*_*ij*_ thereby represent the number of KO's present in pathway *i* in sample *j*. To account for the different pathways sizes, *pm*_*ij*_ was normalized with respect to the number of KO's per pathway *i*.

Clustering analysis is performed using affinity propagation, which is a graph based approach (Frey and Dueck, 2007; Bodenhofer et al., 2011). Pearson correlation was frequently chosen as the final similarity measure and Bray-Curtis similarity in few cases. A general work-flow to assess the most suitable number of clusters is started with high exemplar preferences values, which led to a very large number of clusters. Application of agglomerative clustering on the resulting affinity propagation clusters using the R-package apcluster (Bodenhofer et al., 2011), allowed an inspection of the corresponding dendrogram (Figure S1). Therefore, a cutoff manually decided and affinity propagation rerun repeatedly to achieve the desirable number of clusters.

### 2.8. Feature Selection

The R package Boruta (Kursa and Rudnicki, 2010) was used to obtain a reliable ranking of feature importance and to select only discriminative features for different classification tasks. This algorithm is a wrapper around Random Forest (Breiman, 2001) that performs randomization tests. Features with confidence of importance above 0.99 (the default value in Boruta) were treated as informative. Also the maximal number of importance source runs was increased to 2000 and in some cases to 5000. As the input one of the 75 × *z* feature matrices described above (where 75 corresponds to the number of samples) were used, with *z* varying from around 1016 to 228.256 features depending on the matrix. For example, when summing up all KO abundances per genus the resulting matrix is 75 × 1016. On the other hand, when using KO-genus combinations as features, the matrix extended to 75 × 228256 after filtering. For supervised machine learning, apart from an input feature matrix *X* also a response vector *Y* is used. Here we used prior knowledge of the samples and constructed a response vector based on red or white wine varieties (two classes) or the individual grape varieties (three classes).

### 2.9. Computational Validation

#### 2.9.1. Validation of KEGG Functional Annotation With MetaDraft

As only around 50% of the genes can be mapped to KO's (see Table S1) when analyzing kefir isolates, it is unclear how much information will be lost by mapping compared to just using all genetic information. We therefore created template models for selected KEGG pathways and then used MetaDraft (See section S7; Figure S24) to determine genes that are present in an organism. For a given pathway, all reactions were retrieved along with their corresponding genes that are found in organisms belonging to the phylum *Firmicutes* using the Python package BioServices (Cokelaer et al., 2013). Within MetaDraft, the AutoGraph method (Notebaart et al., 2006) is used, which is a sequence based orthology approach, independent of functional annotation. It is therefore suitable to serve as an independent method to validate the results obtained using KO's.

#### 2.9.2. Validation Computational Findings in Metagenomics

In metagenomics, a computational validation perform using 16S-rRNA reconstruction and binning, which aims to reach the species level of taxonomy. Therefore, it provides extra confidence for the hypothesis generated with the basic computational pipeline on genus level. Moreover, an extra computational validation performed on the concluding results from pathways enrichment analysis on LAB comparison. By removing all close identical sequences (below 99% amino acid similarity) from metagenome samples of reconstructed bins and complete isolate genomes of interest (*L. plantarum*), for example potential exclusive contribution of the high PTS of *L. plantarum* can be determined. Therefore, prediction of an accurate shift of functional potential of the community induced by a single species can be identified.

### 2.10. Assessing Motility of *Acetobacter*

Motility of *Acetobacter* was tested on MRS/whey agar (26 g MRS broth from OXOID, 16 g agar, 500 ml water and 500 ml kefir whey, 48 h fermentation). The plates were incubated for 3 to 4 days at 30°C. Motility was regarded as positive when the cultures spread into the agar and around the spotted colony. Growth only at the spotted area was rated negative. Motility was observed after already 1 day for all four *Acetobacter* isolates. Growth on YPDA for up to 4 days at 30°C revealed no motility.

## 3. Results

### 3.1. Grouping of Genera Based on Presence of KO's

**Figure 2:**
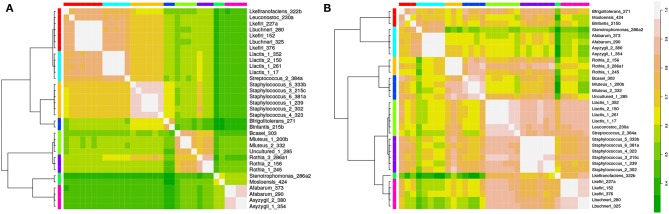
Isolates of the kefir consortium can be grouped using annotated genomes. In **(A)** species are clustered based on presence and absence of KO's yielding eight different clusters. A similar result is obtained when species are clustered based on pathway enrichment i.e., the number of KO's present per pathway **(B)**.

We isolated and sequenced 33 organisms from kefir communities (see section 2.1 for details). To identify discriminative factors between species, we first focused only on the presence and absence of KO's per species and cluster the species based on the KO content using affinity propagation. Hierarchical clustering on top of this result identified eight distinct clusters that separate and in some cases subdivide the genera of *Lactobacilli, Lactococci, Rothia, Acetobacter, Staphylococci* and *Micrococci* (Figure 2). see section 2.7 and Figure S1 for details. This result shows that the KO content alone already has discriminative power and can also lead to non-trivial results, as not only organisms of the same genus group together but also organisms of different genera. The interpretation of the results is, however, not straightforward as the molecular functions assigned to the KO's cannot easily be translated into predictions about physiological characteristics that distinguish the clusters. Therefore, further analyses is required, as described below.

### 3.2. KEGG Pathway Coverage Discriminates Two Groups of *Lactobacilli*

To understand the clustering results better, we mapped the KO's to the level of KEGG pathways and calculated pathway coverage (which is the number of KO's present in this organism in this pathway divided by the total number of KO's in the pathway, see section 2.7). Pathway coverage was subsequently used as input for another clustering. The resulting hierarchical clustering shown in Figure 2 is similar to the one obtained based only on KO presence, except for the *Lactobacilli*. Whereas, these form a single cluster in the previous dendrogram, they are distributed over two clearly separated clusters when using pathway coverage.

**Figure 3:**
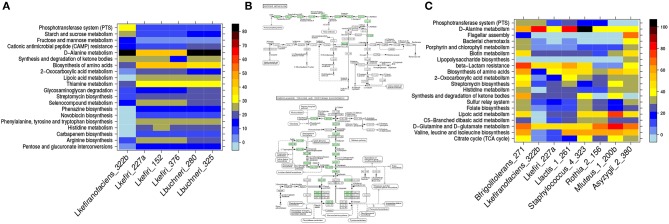
Pathway enrichment reveals differences between organisms within one cluster and between clusters. In **(A)** the six *lactobacilli* isolated from the kefir consortium are compared regarding their pathway coverage. We show the KEGG pathways that have the highest standard deviation based on their pathway coverage. The organisms differ significantly in coverage of e.g., histidine, phenylalanine, tryptophan, and tyrosine metabolism. These pathways are absent in *L. kefiranofaciens* but present in all other *lactobacilli* **(B)**; green means that a KO is present associated with the respective reaction. As there are no KO's found in the maps for *L. kefiranofaciens*, the maps are not shown). In **(C)** the representatives of each cluster (determined by affinity propagation) are compared and the pathways are listed that show the highest standard deviation regarding their pathway coverage. *Acetobacter* (Asyzygii_2_380) have the highest enrichment for both flagella assembly and chemotaxis (discussed in the main text).

To identify the pathways that discriminate the two groups of *Lactobacilli*, we determined all pathways that have a high standard deviation with respect to their coverage. They are shown in Figure 3. The most notable differences are associated with amino acid metabolism: In *L. kefiranofaciens*, histidine, phenylalanine, tryptophan and tyrosine metabolism is completely absent while the remaining *Lactobacilli* all have KO's associated with the synthesis pathways for these amino acids. Conversely, *L. kefiranofaciens* has 27 entries on the phosphotransferase system (PTS) pathway map, whereas the remaining *Lactobacilli* have at most 7 KO's on this map (Figure S2).

### 3.3. Identifying Discriminating Signaling Pathways and Structural Components

This method is not restricted to metabolism but can also make predictions about structural and signaling components represented in KEGG pathways. By identifying the pathways that show the highest standard deviation with respect to their coverage between a representative of each of the clusters, we found that only *Acetobacter* has KO's associated with flagella assembly (Figure 3). They also have the highest pathway coverage for bacterial chemotaxis (Figure 3) which is related to oxygen sensing. Since they are strict aerobes (Sievers and Swings, 2015) both observations would be in agreement with the hypothesis that they use chemotaxis to move on oxygen gradients, and possibly also on gradients of their carbon- and energy source. The presence of flagella in *Acetobacter* was experimentally confirmed (see section 2.9).

### 3.4. Results of KO Annotation Are Consistent With Systematic Pathway Reconstruction

These analyses show that it is possible to create hypotheses about metabolic capacities and structural properties in a fast manner using annotated genomes, in this case annotated with KO's. As only around 50% of the coding sequences can be mapped to KO's (Table S1), there is the possibility that important reactions which do not have KO's associated with them are missed. Therefore, we confirmed the results shown in Figure 3 using an approach that does not rely on KO's but uses only sequence information. For the KEGG maps containing histidine and phenylalanine, tyrosine and tryptophane synthesis pathways, respectively, we created stoichiometric models by retrieving all genes associated with reactions in the respective pathways that belong to organisms of the phylum *Firmicutes* which also covers the genus *Lactobacillus*. Subsequently, InParanoid (O'brien et al., 2005) was used to find orthologs in sequences of the kefir isolates, the corresponding reactions were identified and compared to the reactions associated with present KO's. The results obtained in this way are consistent with the BlastKoala output (Figures S3, S4, and see section 2.9 for details), however, the analysis is far more time-consuming than running BlastKoala even if only these two pathways are considered.

### 3.5. Dynamics of Genera in Wine Fermentations

**Figure 4:**
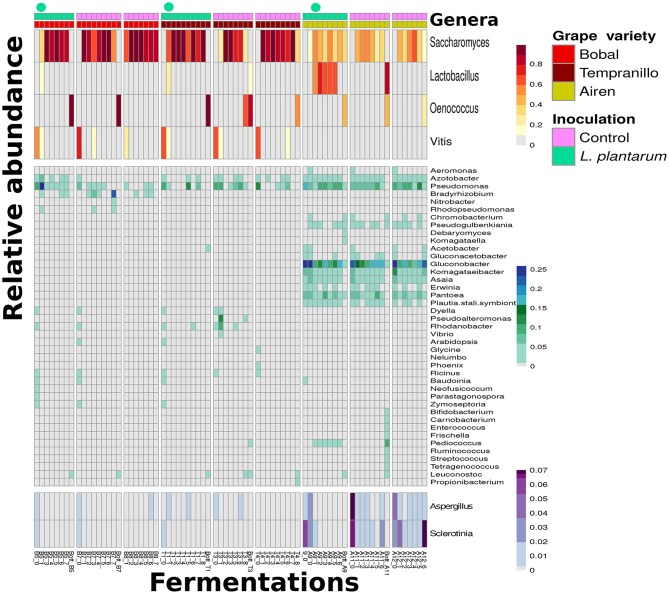
Three types of wine fermentations of grape varieties, Bobal, Tempranillo and Airen indicated by the color bars on top of the figure, the samples are in chronological order of fermentation from left to right. The top color bars and circles indicate the *L. plantarum* inoculations compared to the control fermentations. The Figure shows an overview with abundance of genera in the different wine fermentations, as derived from taxonomic annotation of the metagenome sequences. Only the genera with the highest standard deviation of their abundance across the fermentation are kept. (Top) contain the highest abundance genera, while the (Bottom) is selected specifically for visualization of *Aspergillus* and *Sclerotinia*.

The metagenome of each sample was assembled into contigs and scaffolds (see section 2.6). The open reading frames (ORF's) on these sequences were identified and annotated with KO's using GhostKoala. An overview of the dynamics of abundances of genera was obtained by summing the KO coverage, i.e., the number of reads mapped to the ORF corresponding to the KO, per genus, in each of the samples (Figure 4). Although our basic computational pipeline aims to explore the functional potential of the community, in metagenomics the overview of abundance dynamics can be obtain without extra workload. The table of genera abundances was normalized, and genera with a high standard deviation of abundance across the samples were kept (see section 2.7). A few notable patterns appeared. Firstly, the *Lactobacillus* genus is highly abundant in the samples inoculated with *L. plantarum*. However, the abundance of *Lactobacillus* diminished in time when inoculated in the two red grape varieties, Bobal and Tempranillo, whereas in the white grape variety, Airen, it was highly abundant and the abundance increased during the fermentation. Furthermore, *Lactobacillus* was also present in the Airen controls, in contrast to the control fermentations of the red varieties. Secondly, the abundance of *Lactobacillus* in the Airen variety seems to correlate negatively with the abundance of two genera (*Aspergillus* and *Sclerotinia*), which are spoilage molds. Thirdly, the abundance of *Lactobacillus* is positively correlated with multiple genera such as *Pediococcus, Enterococcus, Oenococcus* (see Figure 4). Fourthly, some genera are present in fermentations of all three grape varieties, like *Pseudomonas, Azotobacter, Vitis* and *Saccharomyces*. Fifthly, some genera occur in fermentations of one variety only, such as *Pantoea* and *Gluconobacter* in Airen, *Dyella* and *Rhodanobacter* in Tempranillo, and *Bradyrhizobium* and *Acetobacter* in Bobal (see section S5; Figures S17, S19, S20, for a systematic investigation of discriminative genera and the corresponding pathways for each wine variety). Finally, the observation of *Saccharomyces* and *Vitis* (grape) DNA is in agreement with the prior knowledge that during the alcoholic fermentation *Saccharomyces* abundance is high and that grape skins are only added at the start of the red wine fermentations and not in the white wine fermentations.

### 3.6. Clustering of Samples Based on KO Abundance in Genera

**Figure 5:**
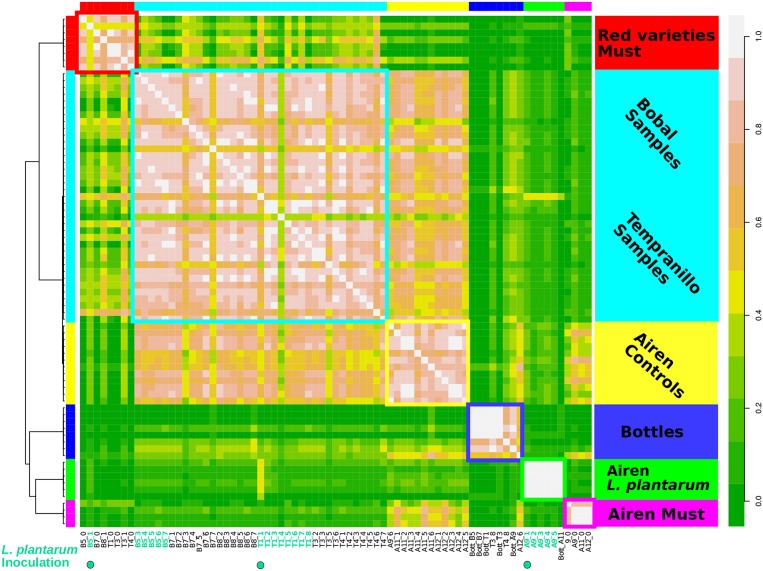
Metagenomics samples of wine fermentation grouped with high resolution using annotated metagenomes. Samples are clustered based on the KO's abundance yielding six different clusters. Left color bar and squares indicates the samples of individual clusters. Similarly inner right side bar legend describe the big majority of the samples. The outer right side bar indicates coloring of the correlation. The resulting groups reveal three discriminative modes.

The mapped data were used to create a table of the KO abundances per genus, which increases the feature space substantially relative to summing these numbers per genus as done above. The samples were clustered using affinity propagation on the Pearson correlation matrix of this table (see section 2.7). This resulted in a high resolution grouping of samples (Figure 5), evidently better than when using reconstructed small subunit (SSU) rRNA abundances (see Figure S10). The microbiomes of the red and white grape varieties could be distinguished, as well as three different stages of fermentation separating the samples of the initial grape must phase, the samples during fermentation, and bottled or final samples of the time series. Finally, the samples of the Airen variety inoculated with *L. plantarum* formed a highly correlated separate cluster. The robustness of the clustering was tested by removing major genera (*Lactobacillus, Oenococcus* and *Saccharomyces*) and a potential artifact (*Vitis*) from the data and reapplying the clustering. The main groups remained essentially unchanged after this procedure (Figure S15).

### 3.7. *L. plantarum* Has the Highest PTS Potential Among the Community

**Figure 6:**
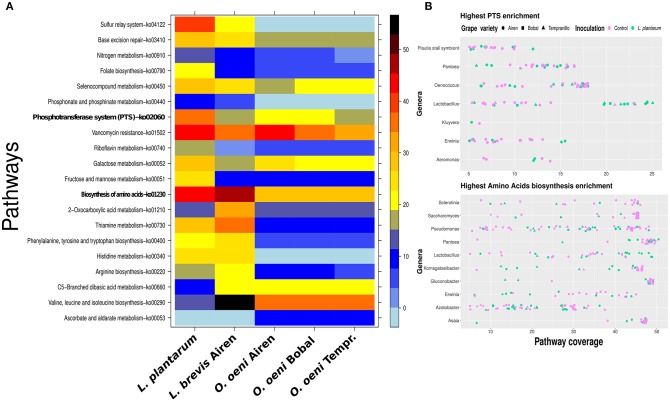
Pathway enrichment reveals differences between the major malolactic fermentors. In **(A)**, a comparison between *L. plantarum, O. oeni* bins and *L. brevis* bin. The right side color bar visualizes the percentage of the coverage of each pathway. In **(B)**, the top ten genera on pathway coverange for PTS (top panel) and amino acids biosynthesis (bottom panel). Only samples with more than 5% coverage are shown, which results in seven genera visualized in the top panel **(B)**.

To confirm that the *Lactobacillus* genus pattern identified so far is indeed the result of the added *L. plantarum* MW-1 strain, we applied a 16S-rRNA reconstruction and binning (see sections S2–S4; Figures S8, S9, S11–S14). As a result we obtained a reconstruction of 16S-rRNA genes of *L. plantarum*. Moreover, the *L. plantarum* draft genome was successfully binned with a high completeness score. Using a few well reconstructed genomes from the binning process, we demonstrate the potential usage of our method also on metagenomic bins. We compared the *L. plantarum* isolate strain with the reconstructed *Lactobacillus brevis* genome bin from the Airen fermentations and the three reconstructed *Oenococcus oeni* genome bins from each variety of grape. The comparison revealed that the *L. plantarum* and *L. brevis* bins had a higher metabolic potential than the three *Oenococcus* bins, especially with regard to amino acid metabolism, PTS and sulfur relay system KEGG pathways (see Figure 6A). Using metagenomic assembly annotations the coverage of *Lactobacilli* PTS stood out when *L. plantarum* was present in the fermentations. (see Figure 6B top). The same effect was observed for genes mapped to amino acid metabolism. Moreover, in addition to *Saccharomyces, Pantoea, Komagataeibacter, Gluconobacter, Erwinia*, and *Asaia* were found to be in the top ten genera with high coverage of amino acid metabolism (Figure 6B bottom). Interestingly, Boruta feature selection analysis assigns the latter five genera as discriminative for Airen against Bobal and Tempranillo (Figure S16).

## 4. Discussion

The examples demonstrating computational analysis on functional and metabolic level show that it is possible to characterize organisms or samples based on KO annotation of genomes, and that hypotheses concerning the physiology and roles of organisms can be derived. This approach is especially useful when studying complex communities. It aims at grouping and contrasting of species by a global comparison of functions. It thereby provides evidence for groups of organisms that might play similar roles, or points to their differences and putative specific roles that they might play in a community. Our computational pipeline can be used in several ways in the research of microbial communities.

When genome sequences of individual community members are available, they can be easily characterized in terms of their functional potential. This is particularly relevant for communities that are not well described. As an example, the *Acetobacter* species stood out among the kefir isolates by the fact that they possess structural genes for the assembly of flagella, as well as a chemotaxis signaling system possibly involved in oxygen sensing Figure 3. Since their motility was confirmed experimentally, these observations suggest an important role for chemotaxis of this species in kefir. Indeed, *Acetobacter* is mostly present in kefir milk, and less in the semi-solid grains, which is in accordance with this hypothesis (Marsh et al., 2013).

Another important observation was that *L. kefiranofaciens*, a dominant organism in kefir (Walsh et al., 2016), stands out among the *Lactobacilli* because of the absence of biosynthesis pathways for a number of amino acids. This species will therefore most likely have several amino acid auxotrophies. Hence, the organism will depend on free amino acids and peptides in milk, which can be present in fresh milk, are released by extracellular enzymatic degradation of milk protein or are produced by other organisms. Whichever way, these auxotrophies will play an important role in the ecology of kefir fermentation.

One should, however, keep in mind that the characterization only concerns genotypic potential. Whether and under which conditions the same genotypic potential also results in identical phenotypes will have to be examined in experiments. We anticipate that the absence of a pathway is more conclusive than its presence as it is most likely context and media dependent whether genes of a pathway are expressed. We strongly believe that this approach provides more insights than a clustering based on gapfilled genome-scale stoichiometric models. To accurately close gaps in pathways one would have to determine an organism-specific biomass composition and grow the individual species under several different conditions to e.g., identify auxotrophies and carbon sources that can be utilized which is very time and resource consuming. It is also very challenging from an experimental point of view as species can be hard to cultivate in isolation. Alternatively, one could also automatically gapfill all the models without experimental validation on a defined medium but then one might miss auxotrophies that can lead to metabolic interactions and the added value of the gapfilling is more than questionable. The presented method focuses only on the gene-associated reactions avoiding all unnecessary overhead and a fast selection of interesting species that can then be examined further in experiments.

Computational analysis was further applied to metagenome data of wine fermentations to explore the effect of the introduction of a *L. plantarum* strain on community composition and dynamics. Furthermore, the dataset, although limited, also allowed an initial exploration of differences between communities in red and white wine fermentations. Together with the functional annotation GhostKoala provides also taxonomic assignment on genus level, which allows not only the exploration of the functional potential of the community, but also the straightforward investigation of genera abundances dynamics.

Therefore, we readily found evidence to support the hypothesis that successful inoculation of a new species to a community was in the case of wine an effect firstly of medium composition, and may determined by fermentation with skin or without skin. Nevertheless, the effect of microbial community interactions such as competition or collaboration cannot be discarded. The experimental results supported this hypothesis (See section S1; Figures S21, S22). Studies on the closely related species *Lactobacillus hilgardii* and *Pediococcus pentosaceus* indicated that phenolic compounds from grape skins could be involved (García-Ruiz et al., 2009). Therefore, the identification of the mechanism behind the inhibition by phenolic compounds as well as the selection of strains resistant to these could play a key role for the usage of organism other than *O. oeni* for MLF in red wines.

The use of annotated metagenomes allowed a fast overview of the community abundance dynamics, such as time-dependent abundance level per genera, presence of common genera in different microbiomes and identification of unique genera in the microbioomes of grape varieties. In addition, we identified putative positive and negative correlations with *L. plantarum*, suggesting for example that *L. plantarum* may inhibits growth of fungi (*Aspergillus*, and *Sclerotinia*), as has been observed before (Valerio et al., 2009; Tropcheva et al., 2014; Lipińska et al., 2016).

By binning metagenomics data and using these to investigate KEGG pathway enrichment, we showed that *L. plantarum* is highly enriched in PTS transport components compared to the other microorganisms in the wine communities. Only a few other metabolic conversions are exclusively found in *L. plantarum* (Fructoselysine/Glucoselysine → Fructoselysine/Glucoselysine 6-phosphate, N-Acetyl-galactosamine → N-Acetyl-galactosamine 6-phosphate, Galactosamine → Galactosamine 6-phosphate (See section S6; Figure S18). These unique properties could play a role in growth of the community.

The shannon index reveals substantial differences in microbial diversity between the white and the two red varieties (Figure S23). The relative abundance of *S. cerevisiae* reaches up to 90% in the red wine fermentations whereas in the white wine fermentations it reaches up to 60%. Also, *Pantoea, Erwinia* from *Enterobacteriaceae* family and *Asaia, Gluconobacter* and *Komagataeibacter* from *Acetobacteraceae* family are exclusively found in the white wine fermentations. These genera are known to be relevant for wine making (Marzano et al., 2016),(Morgan et al., 2017a), in particular acetic acid bacteria for their capacity to oxidize ethanol to acetic acid (Gomes RJ, 2018). Yet, their potential function inside wine communities is not fully explored. We have shown that these five genera have high coverage of metabolic pathways involved in amino acid metabolism. Amino acids, together with ammonium salts, are major nitrogen sources present in grapes, and are essential for microbial growth (Waterhouse, 2016). Moreover, the composition of amino acids seems to influence wine aroma (Hernández-Orte et al., 2002) (Styger et al., 2011). Therefore, studies already examined the effect of microorgansims on amino acid composition during AF (*S. cerevisiae* Fairbairn et al., 2017) and MLF (*O. oeni* and *L. plantarum* Pozo-Bayón et al., 2005). With this in mind, we suggest that the five genera mentioned above are candidates for future investigation.

## Author Contributions

CM and WG conceived the methodology, wrote the code, and performed the analyses. SB, YK, and KP sequenced the genomes of the kefir microorganisms. MA-K, HS, and SS carried out the sequencing of wine metagenomes. CM and NE carried out the inhibition experiments on *L. plantarum*. CM, WG, DM, and BT wrote the paper.

### Conflict of Interest Statement

MA-K, HS, SS, and NE were employed by the company Christian Hansen A/S. The remaining authors declare that the research was conducted in the absence of any commercial or financial relationships that could be construed as a potential conflict of interest.
