# Supplementary material for: Finding Functional Differences Between Species in a Microbial Community: Case Studies in Wine Fermentation and Kefir Culture
Source: Front Microbiol. 2019 Jun 25;10:1347. doi: 10.3389/fmicb.2019.01347 (PMC6603220; doi:10.3389/fmicb.2019.01347)
Supplement: Supplementary file 1 [file Data_Sheet_1.pdf]

Supplementary Material: Finding Functional Differences Between Species in a Microbial Community: Case Studies in Wine Fermentation and Kefir Culture

1 SUPPLEMENTARY TABLES, DATA AVAILABILITY AND FIGURES

Data, accession numbers and scripts used are available on Github: `https://github.com/SystemsBioinformatics/funciminer`. Shotgun sequencing data are available in the European Nucleotide Archive with accession numbers listed in the Github repository in the file wine_accession.tsv. Genome sequencing data are available at NCBI, under BioProject PRJNA375758.

Table S1 lists the genome sequences of strains isolate from kefir used in this study.

S1 Grape skins inhibit growth of *L. plantarum*

The observation that *L. plantarum* hardly, if at all grows in the red wine fermentations suggests that the skins or compounds originating from the skins inhibit its growth. This hypothesis was tested experimentally. The inhibition of *L. plantarum* by skins was tested in 15 samples encompassing pasteurized juices of eight different grape varieties (four white and four red) with or without skins. Fig. S21 shows that growth of *L. plantarum* is inhibited in the initial stages of fermentation. Six samples are not presented in Fig. S21 because *L. plantarum* did not grow in them. Four of these (two Merlot and two Cabarnet) were, in contrast to the other samples, stored frozen together with their skins, possibly causing inhibitory compounds to leach from the skins. One Merlot sample was thermo-vinificated, a method that is likely to promote leaching of skin compounds into the juice. Finally, one white variety (Malagouzia) shows an inhibition of *L. plantarum* growth without skins and slow growth in the presence of skins (Fig. S22). M&M: For each grape variety we followed 8 fermentations, 4 without skins and 4 with skins. All grape juices were adjusted to pH 3.75. 75g Of grape juice and 15g of skins and seeds were put in a bottle. The bottles were pasteurized at 72 °C for 90 seconds, followed by immediate cooling down to room temperature by placing them into sterile water. 3.4g Of frozen *L. plantarum* MW-1 strain was diluted in 99 ml of sterile water, of which 1 ml aliquots were used to inoculate 2 out of 4 samples of both conditions (skins or no skins). Sampling was carried out on day 0 (the day of inoculation), after 2 hours, on day 1 and on day 3. Samples were plated on Square Petri Dishes on artificial grape juice agar. The plates were incubated at 30 °C, while fermentations were carried out at 25 °C.

S2 Investigation of metagenomes using 16S-rRNA reconstruction

To obtain an overview of communities in wine fermentations and to increase the taxonomic accuracy, we reconstructed the full-length ribosomal (SSU or 16S-rRNA) genes. Afterwards, the SSU's are clustered to OTUs and subsequently used to create a biome table, B, of dimensions $n \times m$, where m is the samples and n is the number of resulting OTU's. The total number of unique OTU's during the fermentation is followed in the three grape varieties and different fermentation tanks (Fig. S8), yielding a total of nine fermentations. The number of OTU's is higher at the initial time points (the grape must samples) and decreases until the end (the bottled samples), which have the least diversity and a smaller total number of OTUs.

WGS	Species name	Mapped genes [%]
PDEW00000000	L.lactis	46.3
NCXG00000000	L.lactis	46.9
NDFJ00000000	L.kefiri	51.1
NDFM00000000	R.dentocariosa	44.6
NCWR00000000	M.luteus	54.3
NCXH00000000	S.haemolyticus	55.7
NDFN00000000	B.drentensis	47.3
NCWS00000000	L.kefiri	51.5
NCWT00000000	L.mesenteroides	60.8
NDFK00000000	S.saccharolyticus	57.5
NCWU00000000	R.dentocariosa	49.0
NCWV00000000	L.lactis	48.2
NCWW00000000	B.simplex	47.4
NCXI00000000	L.parakefiri	52.7
NGUZ00000000	C.tuberculostearicum	52.0
PDEV00000000	R.dentocariosa	47.0
NCWX00000000	S.rhizophila	52.5
NDFL00000000	S.saccharolyticus	56.4
NCWY00000000	B.casei	47.5
NCWZ00000000	L.kefiranofaciens	51.5
NCXJ00000000	S.pasteuri	56.9
NCXA00000000	L.parakefiri	51.9
NCXE00000000	M.luteus	51.2
NCXB00000000	S.hominis	59.9
NCXC00000000	L.lactis	46.1
NDFO00000000	A.ghanensis	54.8
NCXK00000000	A.fabarum	52.7
NCXD00000000	L.kefiri	52.4
NDFP00000000	A.ghanensis	54.7
NDFI00000000	S.saccharolyticus	58.4
NGVM00000000	S.pseudopneumoniae	52.5
NCXF00000000	M.osloensis	62.5

Table S1. WGS identifier, species and the percentage of genes that could be mapped to KO's.

An exploratory data analysis reveals that the microbiome of the white grape variety Airen is very different from the two red grap varieties Bobal and Tempranillo. using non-metric multidimensional scaling (NMDS) and Bray–Curtis dissimilarity on the data of table B groups of samples can be discriminated (Fig. S9 A). The white Airen variety microbiome separates from the two red grape varieties (Fig. S9 C, B). Furthermore, in the same plots the initial grape musts samples (before the fermentation) are always projected close to each other.

The samples were clustered using affinity propagation with Pearson correlation distance calculated from table B. Once more, an evident separation ensues between the white and the two red varieties (Fig. S10 C, B).

SSU reconstructions and taxonomic assignment on species level of SSU genes gave valuable insights, such as the separation between microbiomes on white and red grape varieties, and the exploration of simple dynamics, like the diminishing number of OTU's during fermentation. However, the relative abundance of the OTU's is not precise, due to the uneven depth of sequencing(Fig. S5). Correlations between the dynamic abundance data and, for example, the inoculation of *L. plantarum* in 1 out of the 3 respective time

points could not be distinguished. Moreover, the process of reconstruction is time consuming and heavy in computational requirements.

S3 Reconstructing draft genomes using binning

We apply binning using the MaxBin 2.0 tool (Wu et al., 2015, 2014), which allows a reconstruction of draft genomes. Using these, estimates of the relative abundances of the reconstructed draft genomes can be obtained by counting the number of corresponding sequence reads. Only a small part of the members of the reconstructions were considered good enough, based on the completeness score (which is the fraction of unique marker genes versus of 107 marker genes) and were used for further analysis. In total, the data allowed a reconstruction of 24 draft bacteria genomes with a completeness above 70%. The overview in Fig. S12 shows these draft genomes for fermentations of each grape variety with the corresponding scores.

The three different varieties binned independently by merging beforehand the shotgun samples into three ”super” samples and assembly with IDBA-ud (Peng et al., 2012). To visualize the results we use t-distributed stochastic neighbor embedding (t-SNE) on the pentaoligonucleotide frequencies (Laczny et al., 2014) into three dimensional space (Fig. S11).

The abundance of *Lactobacilli* bins during the different wine fermentations is shown in Fig. S13, top. The *L. plantarum* inoculatiopns can be identified for each grape variety. Interestingly, the abundance of *L. plantarum* diminishes when inoculated in the two red grape varieties, whereas in the white grape variety it remains highly abundant and even increases. Furthermore, *L. plantarum* is present in the Airen control samples, in contrast to the control samples of the red varieties. In addition, another *Lactobacillus* bin was found only in the Airen microbiome with assigned taxonomy of *Lactobacillus brevis*.

S4 L. plantarum correspondence between binning and KO results

To pursue the *L. plantarum* we also annotated its genome, which was produced by isolation and individual sequencing (see material and methods for more details). Figure S13 - bottom shows the presence and absence of *L. plantarum* KOs during the different fermentation periods, the pattern observations are greatly similar to the bin abundance bar plot (Fig. S13 - top). Additionally, with this alternative approach the visualization provides an important advantage. The bars of the Airen control samples show higher similarity in terms of *L. plantarum* KOs than the control samples from the two red varieties. Hence, in Airen microbiome we already found *L. plantarum* and another close related *Lactobacillus* bin. Yet, by looking at all the other genera based on the NCBI taxonomy of Ghostkoala (Fig. S14) is noticeable that multiple genera show higher similarity to the *L. plantarum* KOs.

S5 Feature selection on metagenomics wine microbiome

For the goal of targeted identification of discriminative genera and the corresponding pathways for each variety we exploit the random forest feature selection. Firstly, we apply the selection process to the matrix G and identify fifty-four genera in total (Fig. S16 A). After filtering the genera with low standard deviation the number reduced to ten (Fig. S16 B). This analysis reveals *Gluconobacter*, *Pantoea*, *Komagataeibacter* and *Asaia* abundance to be discriminative for Airen microbiome, *Pseudoalteromonas* for Tempranillo microbiome and *Bradyrhizobium* as well as *Rhodopseudomonas* for Bobal microbiom.

To explore biological implication we continue by mapping KOs to KEGG pathways and create another matrix P, where n now represent the pathway coverage of the KOs (see m&m for details). Afterwards, we apply feature selection once more on the same classification problem. This led to eighty-one pathways that belong to eighteen genera, eleven out of eighteen were also found using the matrix G (Fig. S17). We

found that *Gluconobacter* had many discriminative pathways for the Airen microbiome, for example a high number of amino acid biosynthesis and starch and sucrose metabolism genes. Also genes of glutathione metabolism from *Chromobacterium*, and amino acid (lysine) and pantothenate and CoA biosynthesis genes of *Pseudogulbenkiania* were discriminative. Finally, genes from biotin metabolism of *Asaia* were discriminative. Only a few genera had discriminative pathways for the Tempranillo microbiome such as *Rhodonobacter* and *Dyella*. On the other hand, many pathways from the *Bradyrhizobium* genus, for example involved in bacterial chemotaxis, beta-lactam resistance fatty acid, carbon, sulfur and nitrogen metabolism were discriminative for the Bobal microbiome.

Finally, we investigate the presence of flagellar assembly pathway together with the bacterial chemotaxis pathway(Fig. S20), a combination which could lead to identification of microorganism with potential capability to move and influence the structural properties of the community inside the fermentation tank. We found enrichment on these two pathways at *Pseudomonas* genus, which is present on all three microbiomes and few genera only at Airen microbiome, such as *Gluconobacter*, *Gluconacetobacter*, *Pantoea*.

S6 Comparison of metabolic capacity between L. plantarum and O. oeni.

We already found an interesting pattern of *L. plantarum* abundance between the inoculation tanks, and possible influence when it is highly abundant towards other members of the community. Therefore, We continue the investigation of *L. plantarum* potential influence based on metabolism to the whole community by using our "*in silico* screening" method (Fig. 6 A). In a closer inspection the two *Lactobacilli* have complete histidine pathway in contrast to *Oenoccocus* bins Fig. S19. Moreover, we found that *Pantoea* and *Erwinia* have a high number of PTS genes, but still far lower than the *Lactobacillus* and *Oenococcus* genus (Fig. 6B top).

Finally, to determine if *L. plantarum* high PTS capabilities could influence the potential functional properties of the community, we remove all *L. plantarum* ORFs from four selected samples (first days of four different fermentations from two varieties) and investigate the effect in pathways in terms of presence or absence of reactions. The PTS pathway of the two controls samples shows no change at all after the removal. However, on both inoculation samples we found three sugar uptake conversions (reported in discussion) that disappear after the removal (Fig. S18). Moreover, we identify other exclusive metabolic capability of *L. plantarum*, like two abc transporters: cobalt and nickel, a reaction in glycerophosholpid metabolism, etc. Still, these findings correspond only to the first day of the selected samples. Further exploration reveal a difference in PTS enrichment between the reconstructed *O. oeni* draft genomes, the second species in PTS enrichment and the main malolactic fermenter in wine making. Although, the two *O. oeni* reconstructed from the two red varieties metagenome don't have any of the *L. plantarum* "unique" PTS conversion identified on the time of inoculation. In contrast the *O. oeni* reconstructed from the white variety metagenome has one KO the same with *L. plantarum* PTS (K02744, PTS system, N-acetylgalactosamine-specific IIA component)

S7 MetaDraft

The quantitative analysis and modelling of metabolism is an important tool in the modern systems biologist's toolbox. More specifically, constraint-based modelling as applied to genome scale reconstructions (GSR's) has proven to be useful in elucidating both the fundamental properties of metabolic networks. GSR's also provide a bridge between metabolic function and an organisms 'genomic potential' through the definition of gene-protein-reaction associations (GPR's) (Thiele and Palsson, 2010). In general the process of creating GSR's by hand is a time and labour intensive process. While various automated

pipelines exist to facilitate this process these are mostly geared to the creation of a complete 'working' models and are not easily modified for other purposes (Overbeek et al., 2005; N et al., 2011).

MetaDraft has primarily been developed as a user-friendly, graphical tool to facilitate the generation of draft, stoichiometrically balanced, metabolic networks. Using the AutoGraph method it utilises a sequence based orthology approach (Notebaart et al., 2006) which is independent of any genome specific, functional annotation. Metadraft is available on request from Dr. Brett G. Olivier (b.g.olivier@vu.nl)

S8 Figures

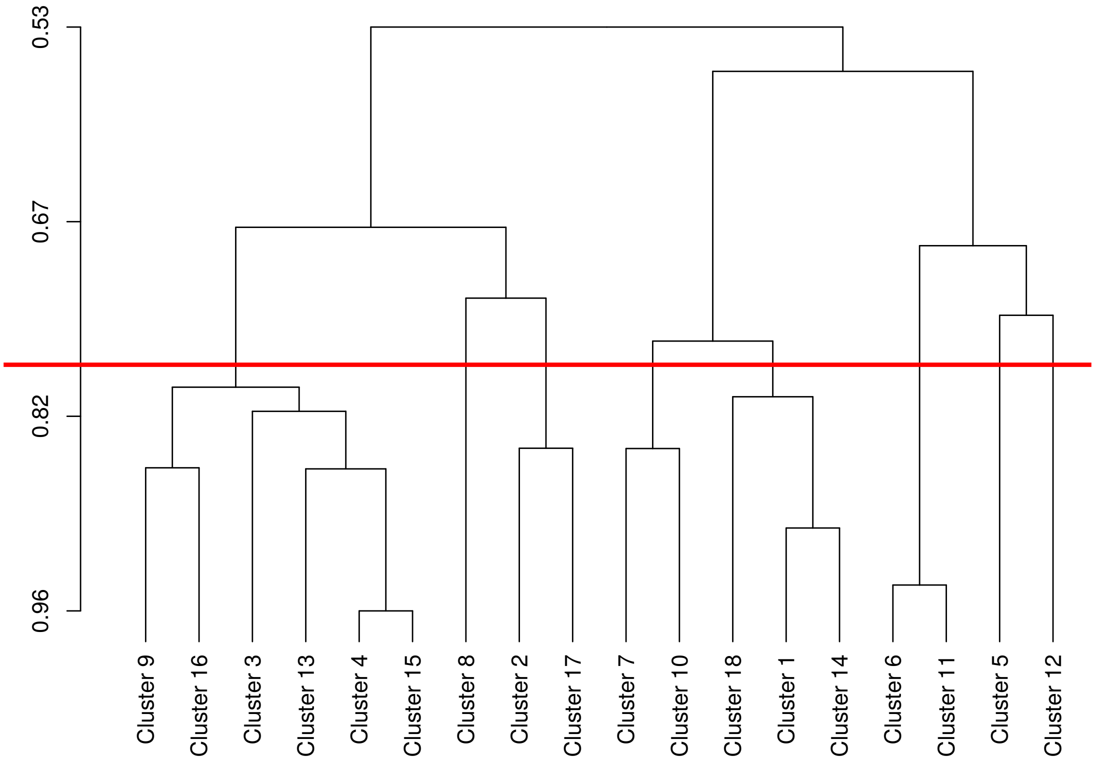

Figure S1. Dendrogram based on exemplar-based agglomerative clustering on top of the result obtained by affinity propagation. The red line indicates the cut-off which leads to eight distinct clusters.

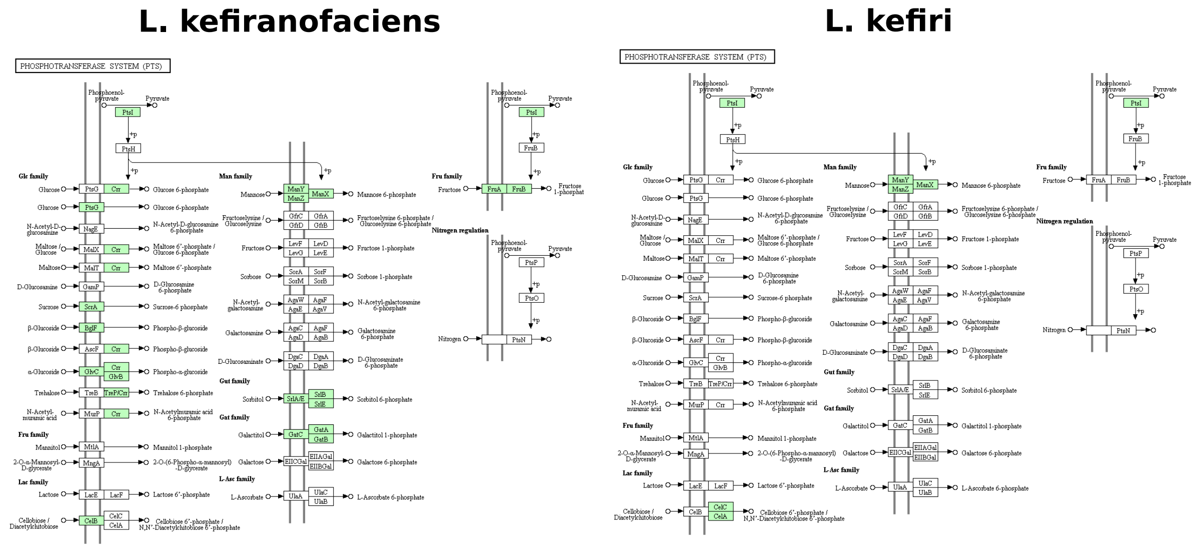

Figure S2. Comparison of *L. kefiranofaciens* and *L. kefiri* regarding the KO coverage of the PTS. Reactions shown in green have a least one KO associated with them.

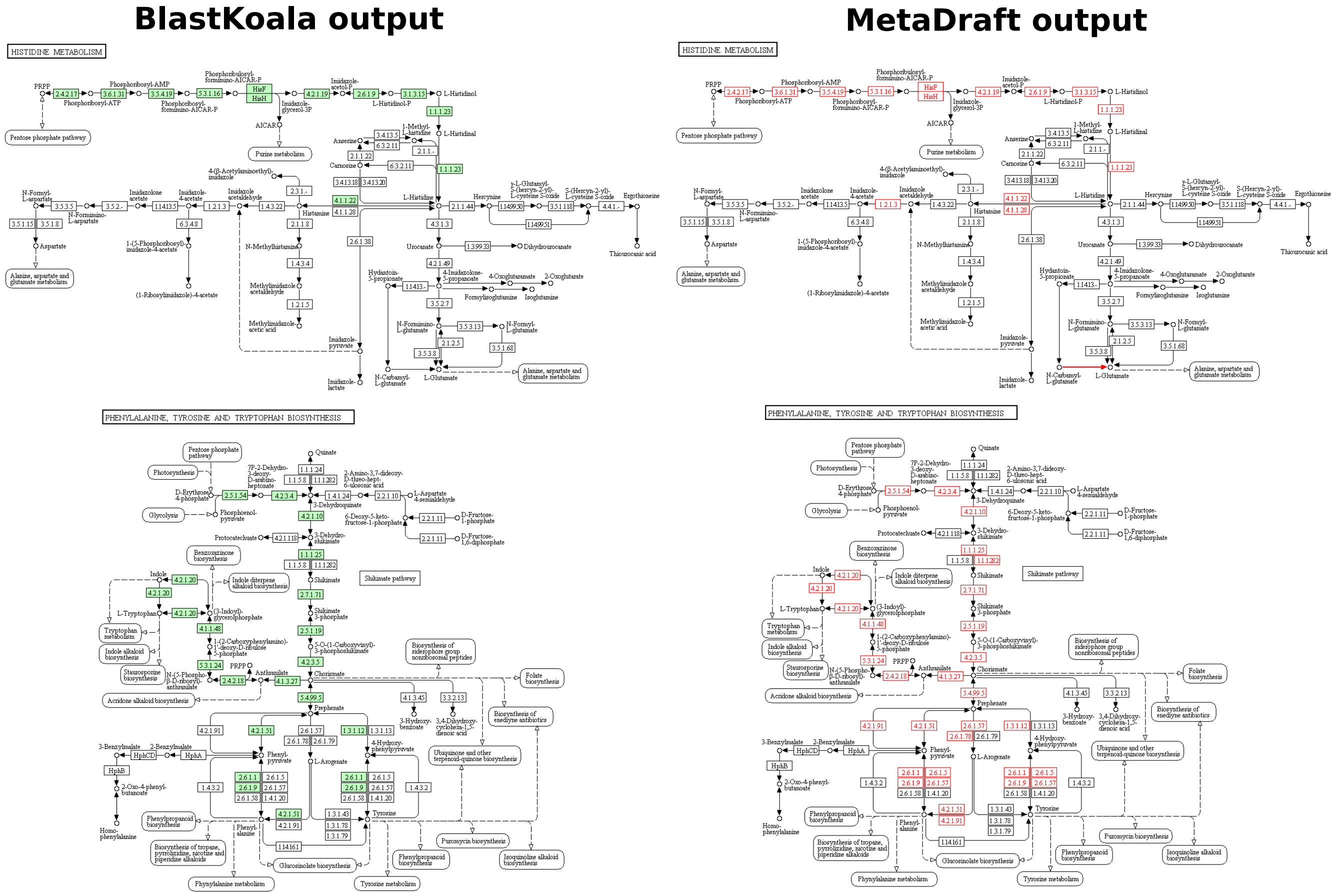

Figure S3. Validation of the BlastKoala output using MetaDraft. The green reactions are associated with at least one KO present in the organism *L. buchneri*, the red ones have at least one gene of an organism of the phylum ”Firmicutes” associated with them.

BlastKoala output **MetaDraft output**

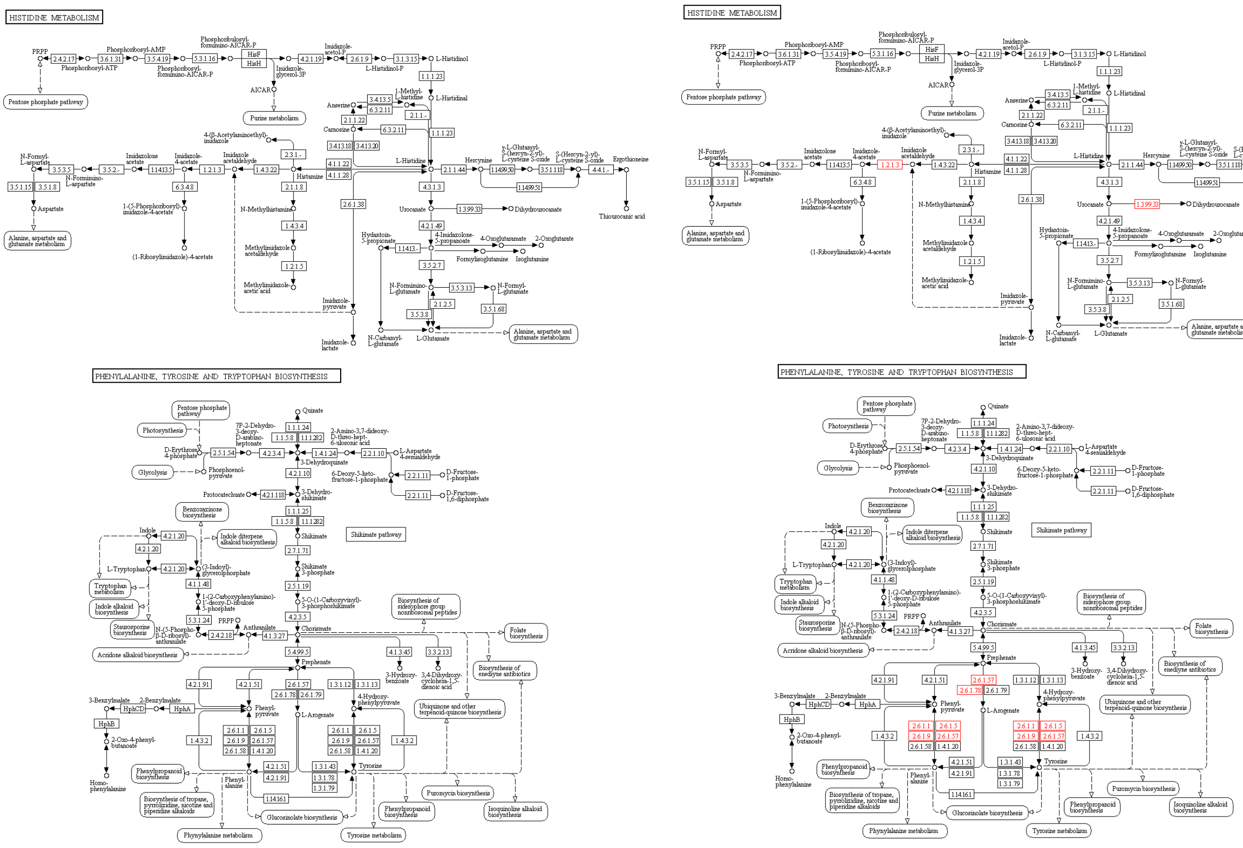

Figure S4. Validation of the BlastKoala output using MetaDraft. There are no KO's in the organism *L. kefiranofaciens*, the red reactions have at least one gene of an organism of the phylum "Firmicutes" associated with them.

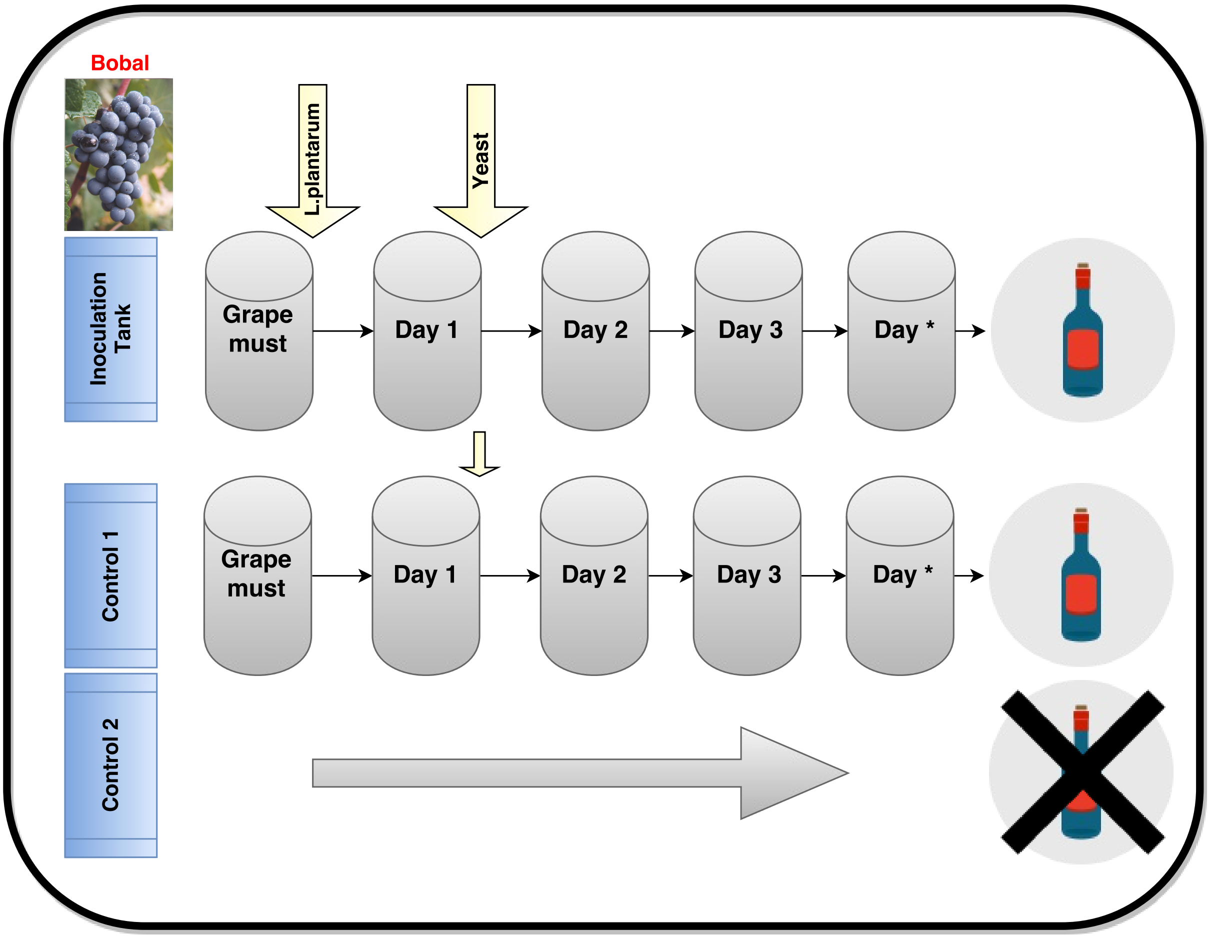

Figure S5. Experimental design of wine fermentation. for each grape variety three fermentation were followed. One of the three was inoculated with *L. plantarum* strain, while the other two not. All three were inoculated with the same *S. cerevisiae*. Samples names are explained in Fig. S6

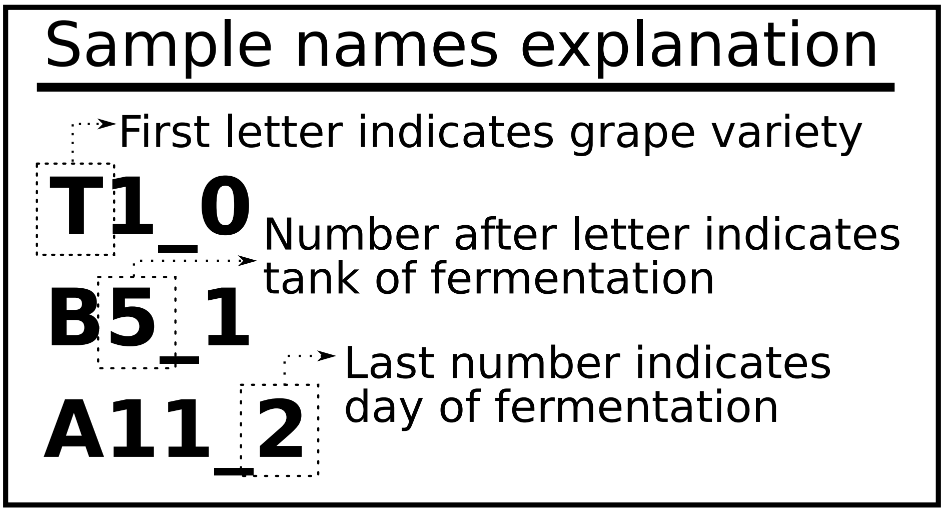

Figure S6. Explanation of sample names of 75 metagenome samples, which correspond to 9 different wine fermentations of the three grape varieties

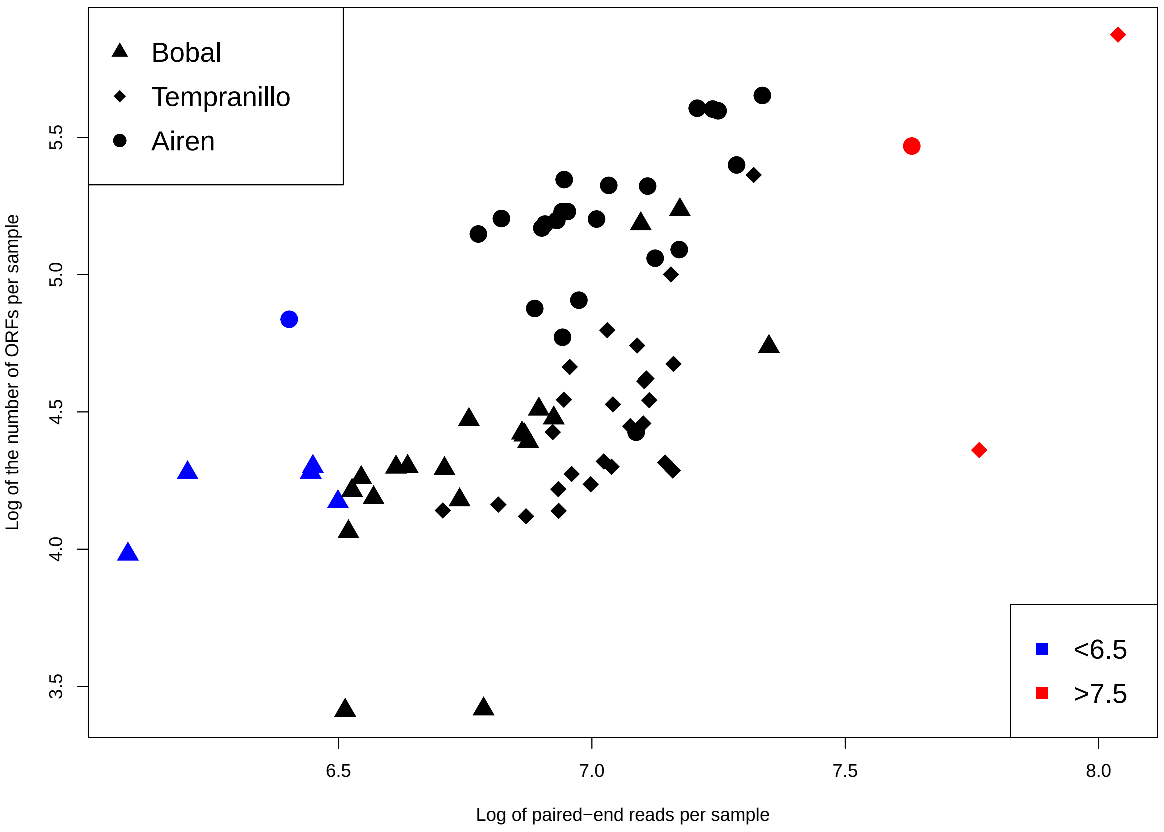

Figure S7. Overview of data size effect. Samples were sequenced at uneven depth. The plot displays the total number of predicted ORFs against the paired - end read count for each sample. Shapes correspond to different grape varieties and colored samples should be considered with caution

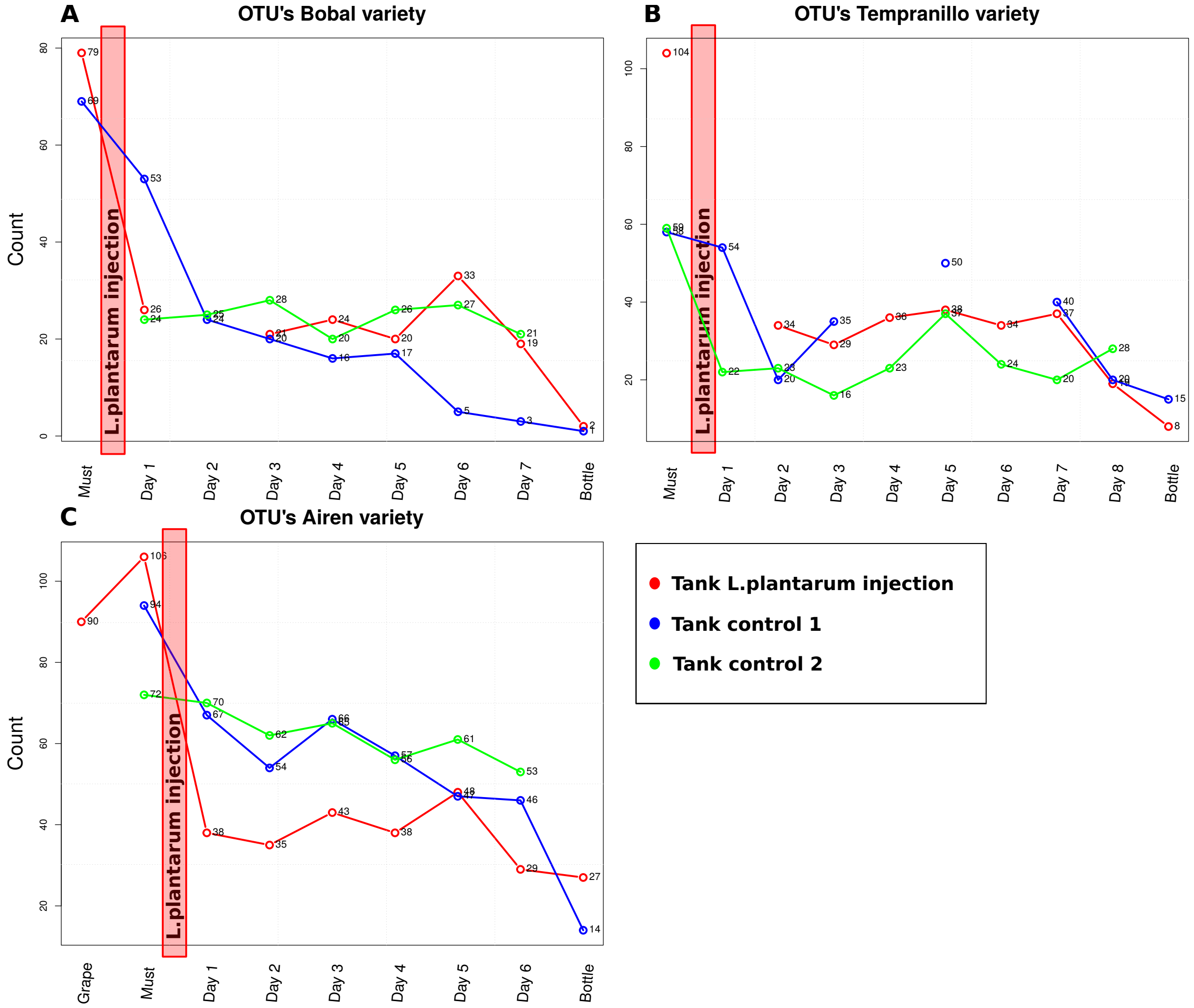

Figure S8. OTU count during wine fermentation. Each plot contains the data for a grape variety. Red: *L. plantarum* inoclulation, with blue and green: two controls. **(A)**: Bobal (red) variety, **(B)**: Tempranillo (red) variety, **(C)**: Airen (white) variety

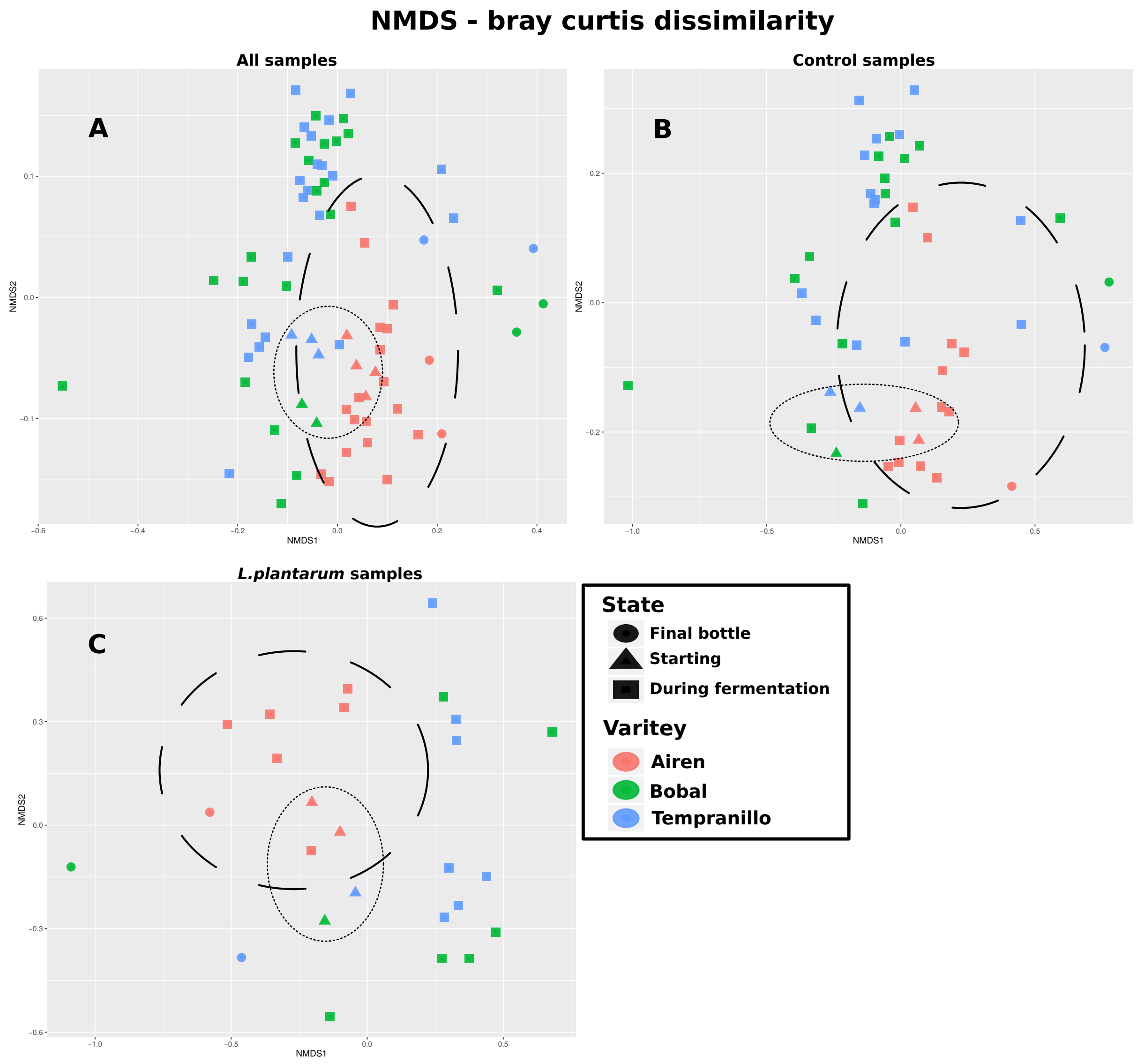

Figure S9. Dimensionality reduction plots with non-metric multidimensional scaling on Bray-Curtis dissimilarity of the OTU table. Red color represents the Airen (white) variety and dashed circles were drawn by hand around them. Triangle shapes represent starting points, grape or must samples and dotted circles were drawn by hand around them. **(A)**: All samples together, **(B)**: The two control fermentation for each variety, **(C)**: The *L. plantarum* inoculated fermentation for each variety.

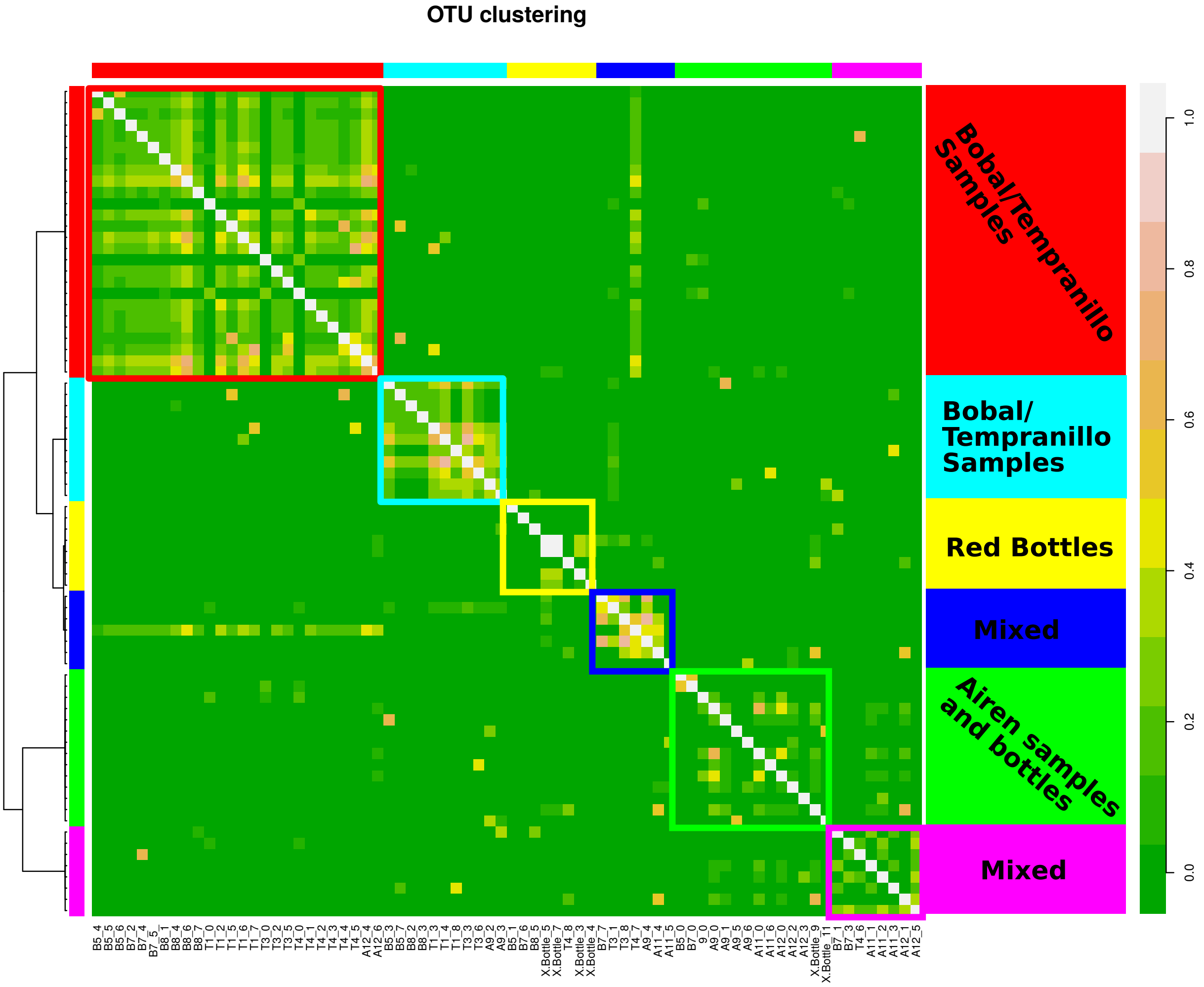

Figure S10. Clustering samples with affinity propagation using Pearson correlation on the OTU table. The number of clusters was predefined to six to be comparable with later clustering results.

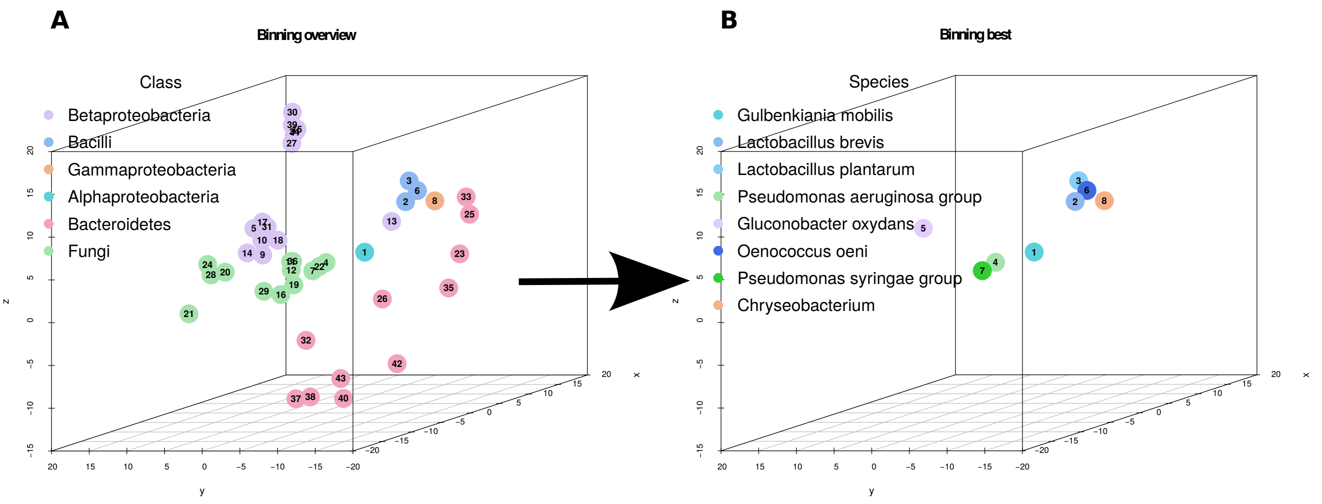

Figure S11. Visualization of binning of Airen microbiome. Dimensionality reduction was performed with t-SNE on pentaoligonucleotide frequencies, and color grouping is based on the results of the maxBin software. Numbers inside the circles represent the highest completeness score, with one been the highest. **(A)**: All bins, assigned on class level taxonomy. **(B)**: Bins with the highest completeness, which are usable for further analysis, assigned on species level taxonomy.

Bobal

Taxonomy	Completeness
Rhodanobacter sp. 115	99.1%
Pseudomonas syringae group	98.1%
Oenococcus oeni	96.3%
Bradyrhizobium sp. BTAi1	95.3%
Propionibacteriaceae	86.0%
Lactobacillus plantarum	78.5%

Tempranillo

Taxonomy	Completeness
Lactobacillus plantarum	99.1%
Rhodanobacter sp. 115	99.1%
Pseudomonas syringae group	97.2%
Oenococcus oeni	95.3%
Pseudoalteromonas haloplanktis	95.3%
Pezizomycotina	70.1%

Airen

Taxonomy	Completeness
Gulbenkiania mobilis	100.0%
Lactobacillus brevis	99.1%
Lactobacillus plantarum	95.3%
Oenococcus oeni	93.5%
Chryseobacterium	92.5%
Pseudomonas aeruginosa group	91.6%
Gluconobacter oxydans	90.7%
Pseudomonas syringae group	85.0%
Pseudomonas syringae group	82.2%
Komagataeibacter hansenii	79.4%
Asaia prunellae	72.9%
Sphingomonas sp. FUKUSWIS1	72.9%

Figure S12. Table with reconstructed bins per grape variety with completeness above 70%.

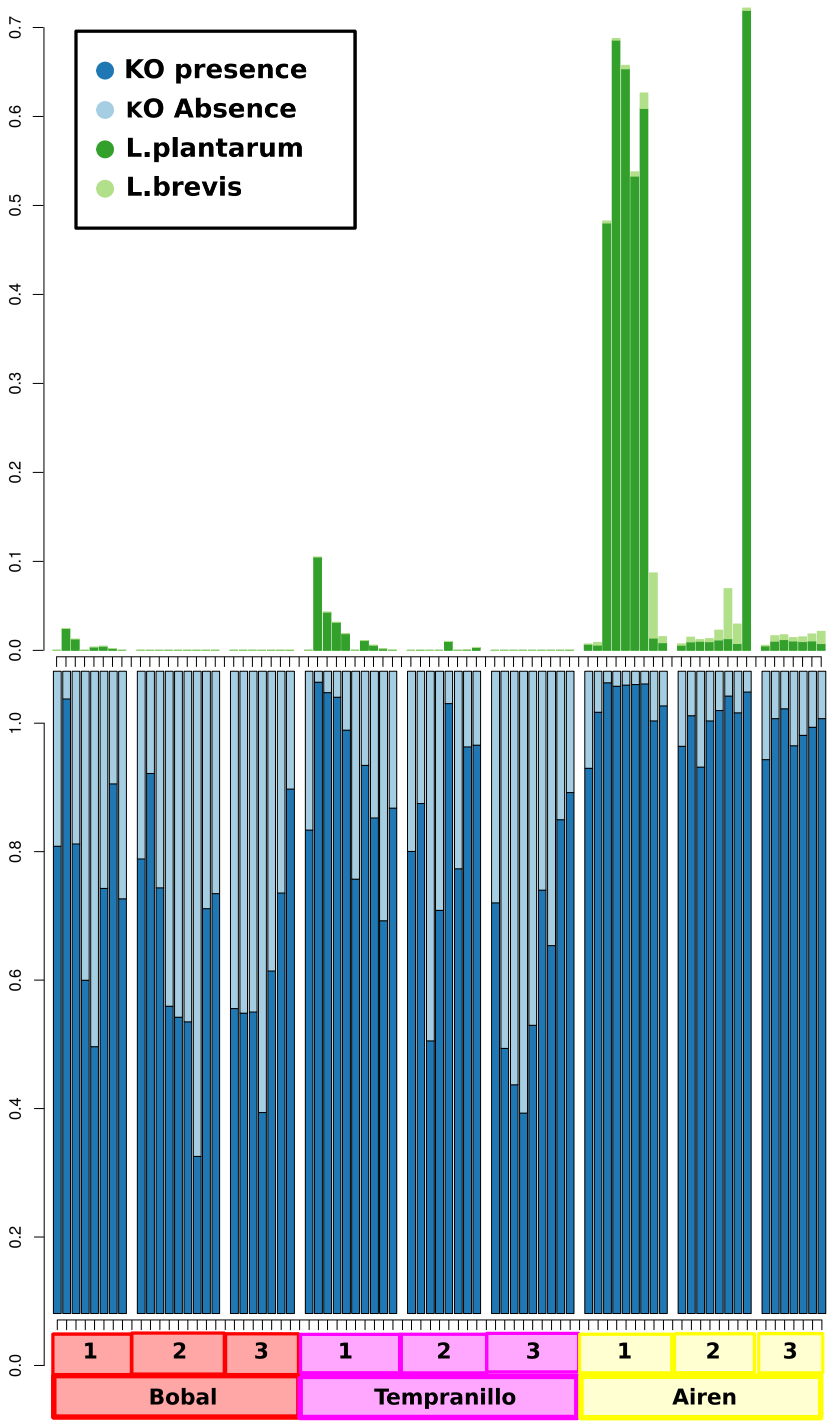

Figure S13. Three types of wine fermentations of grape varieties Bobal, Tempranillo and Airen indicated by the bar below figures, the samples are ordered chronological during fermentation from left to right, the numbers inside legend bars below and above the grapes names bars indicate with 1 the *L. plantarum* inoculation, 2 and 3 are control fermentations. Moreover, the last sample/bar of tanks 1 and 2 is from bottle while for tank 3 is absent for all varieties. Data are derived from the metagenome shotgun sequences Fig. S5. The upper panel shows the relative abundance of *L. plantarum* and its close relative *L. brevis* in wine. Note the high abundance of these bacteria in white wine. The lower panel shows the presence or absence of *L. plantarum* KEGG Orthologs (KO) against each KO profile of the complete community.

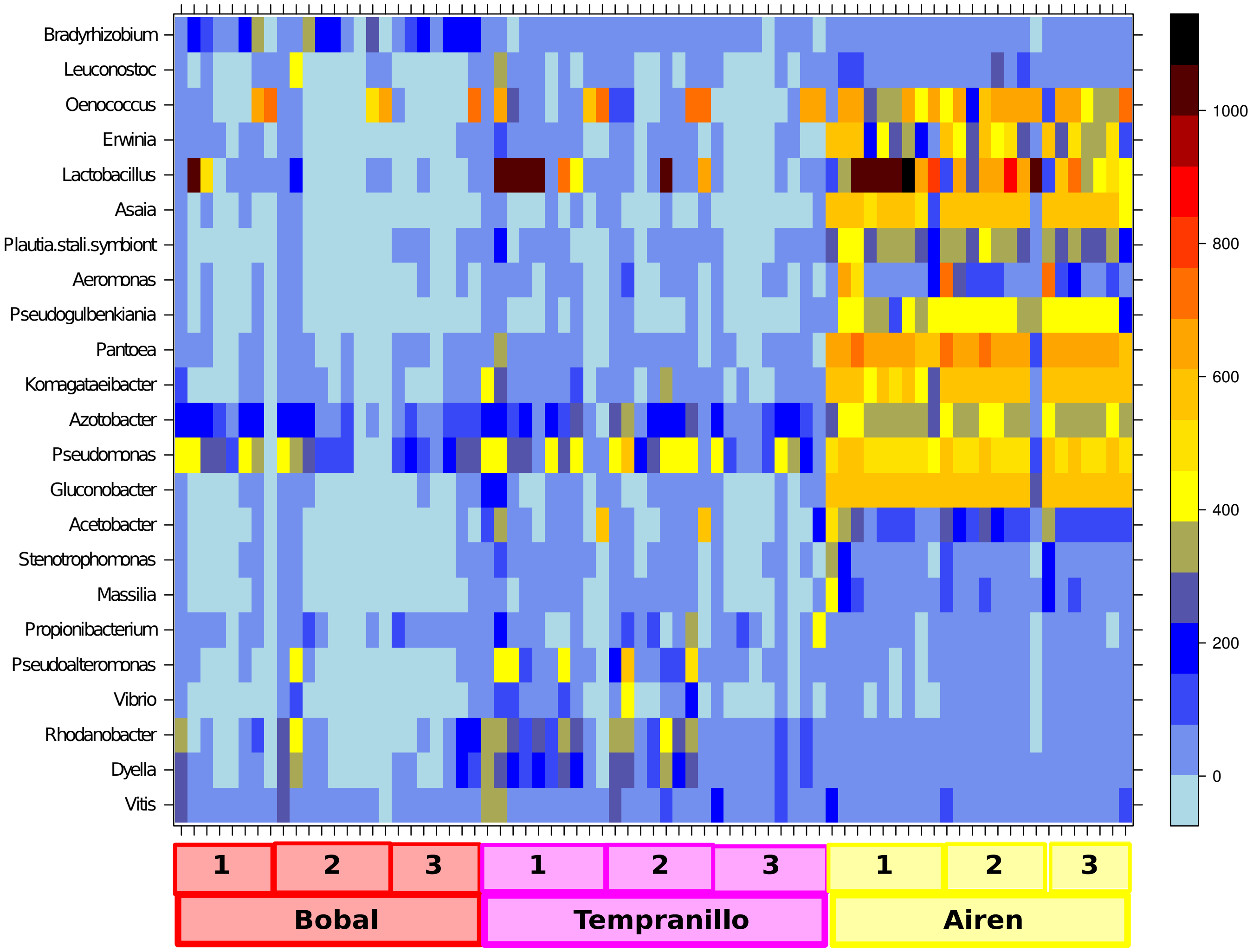

Figure S14. Heatmap of genera with high number of same *L. plantarum* KOs.

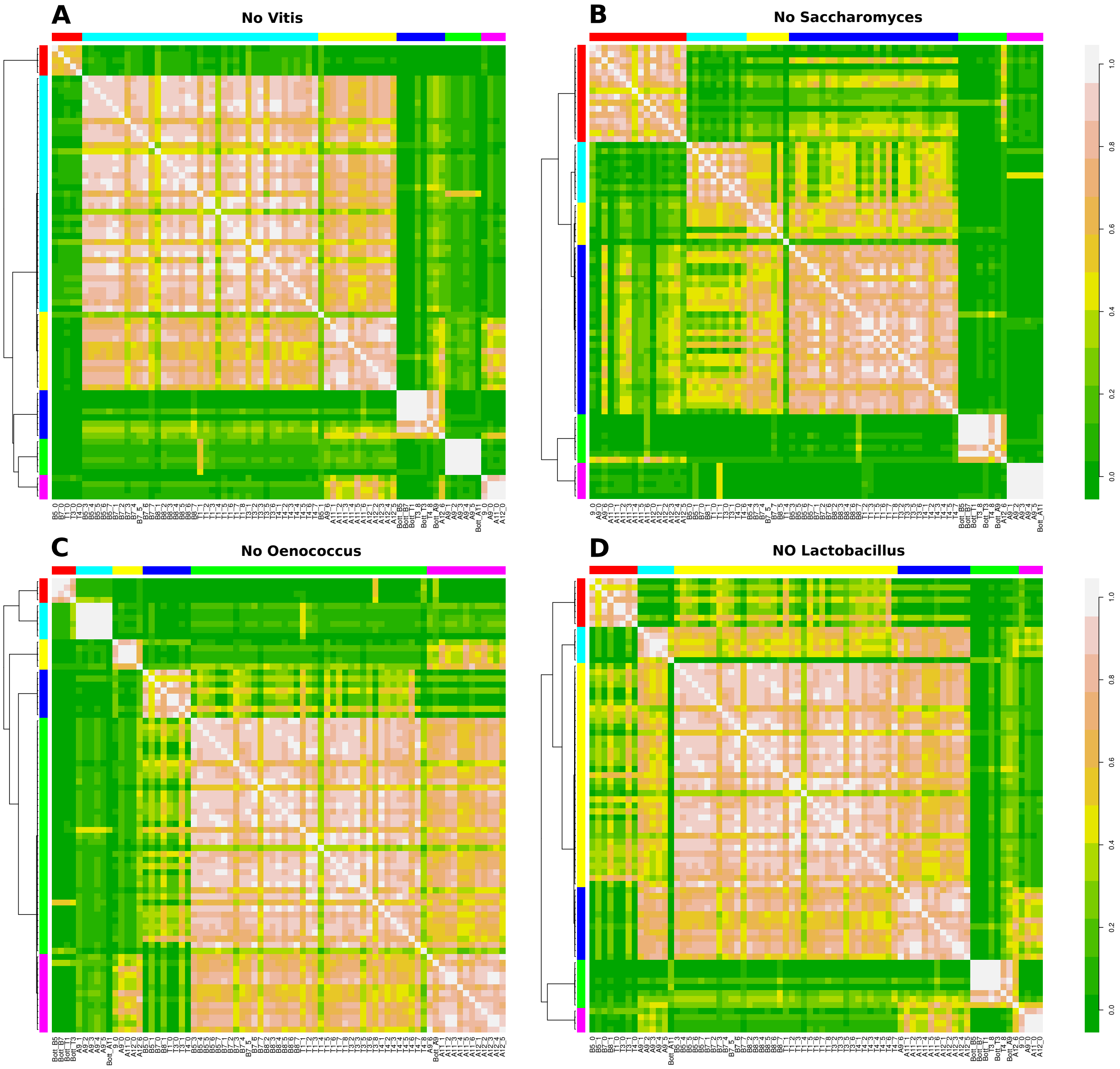

Figure S15. Clustering robustness test. We reapply affinity propagation with a predefined number of six clusters. In each panel we removed major genera from the analysis to see their effect on the clustering result. (**A**): Removed *Vitis* KOs, (**B**): Removed *Saccharomyces* KO's, (**C**): Removed *Oenococcus* KO's, (**D**): Removed *Lactobacillus* KO's.

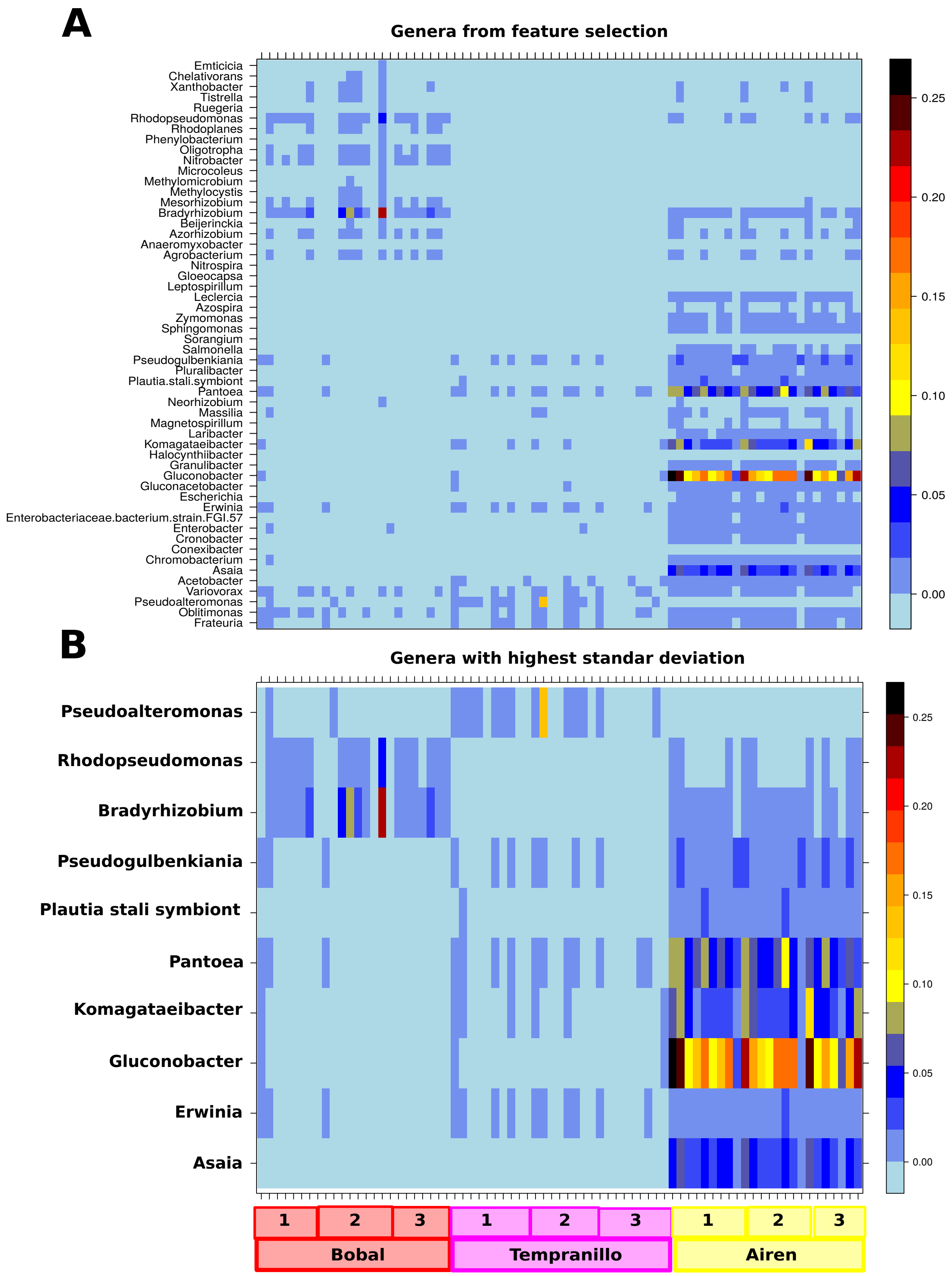

Figure S16. Heatmap with discriminative genera for classification between three grape varieties. **(A)**: Heatmap with all genera, 83 in total. **(B)**: Genera with with low standard deviation on their abundance levels were filtered out, leading to 10 discriminative genera.

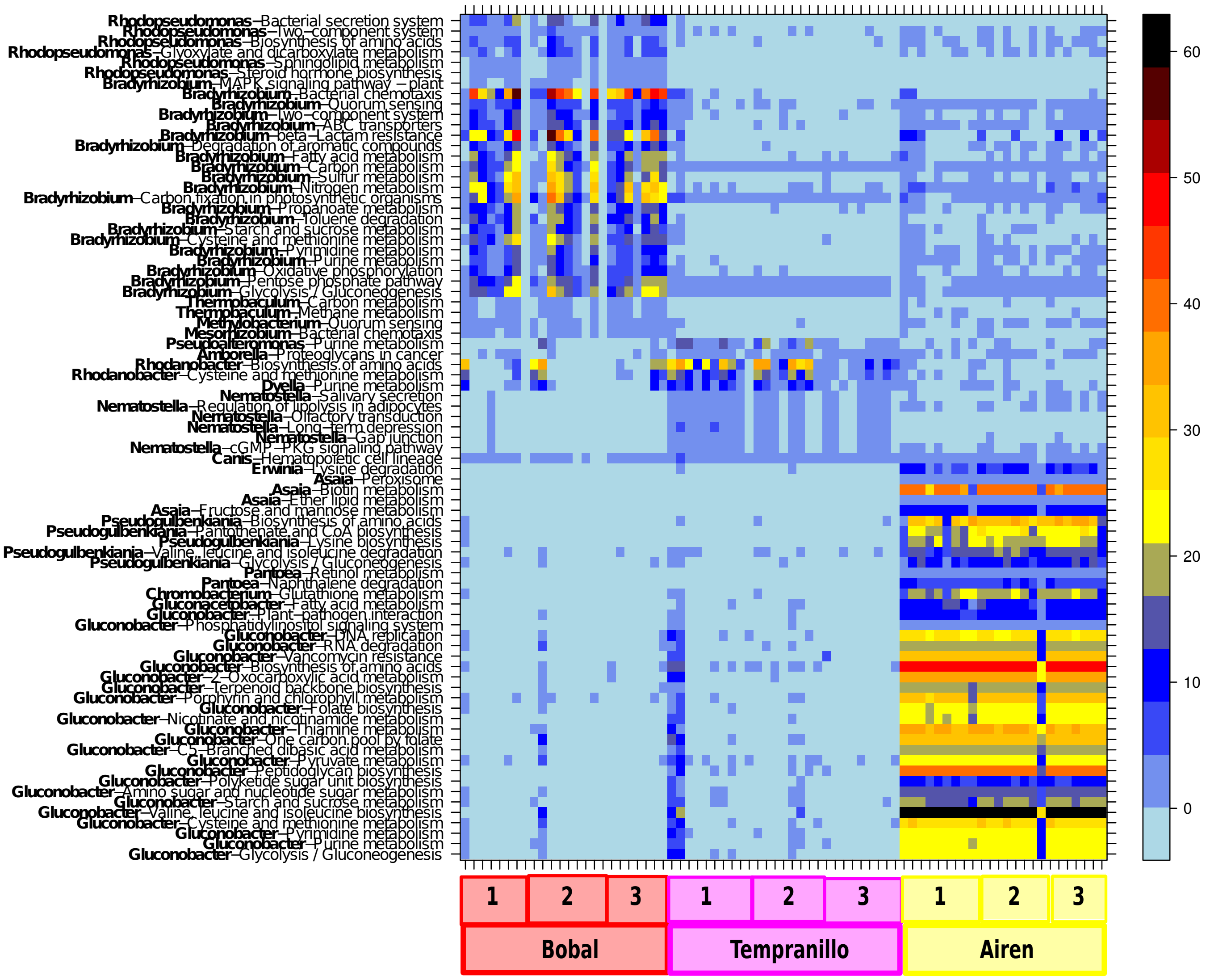

Figure S17. Heatmap with discriminative pathway-genera. Feature selection with random forest was applied on a three class problem (grape varieties) to filter out relevant genera based on pathway enrichment.

Complete community: Day 1, Airen inoculation

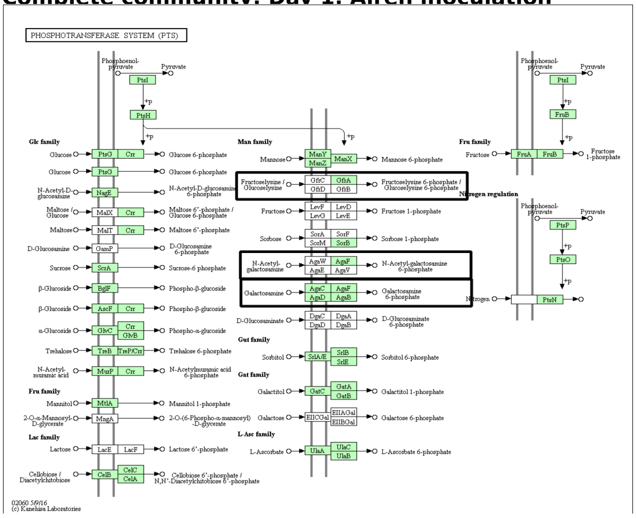

Community without L. plantarum: Day 1, Airen inoculation

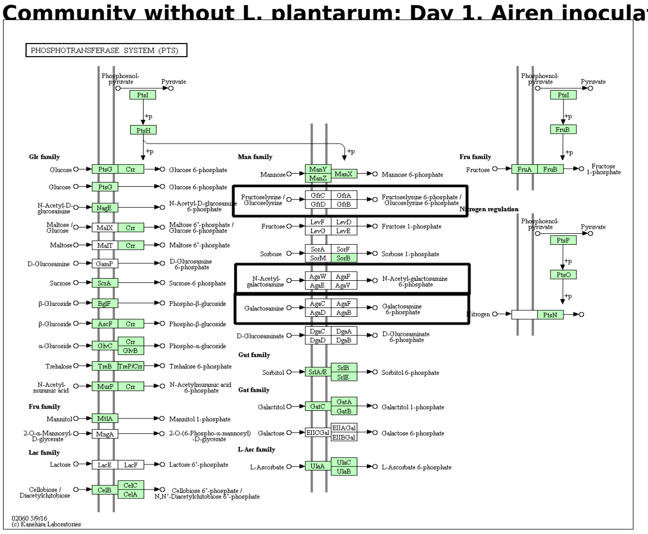

Figure S18. Three PTS systems exclusively provided by *L. plantarum*, found by a comparison of the PTS of the whole community and the PTS of the community without *L. plantarum*. The data for this figure were obtained from the first time point of the Airen inoculation tank.

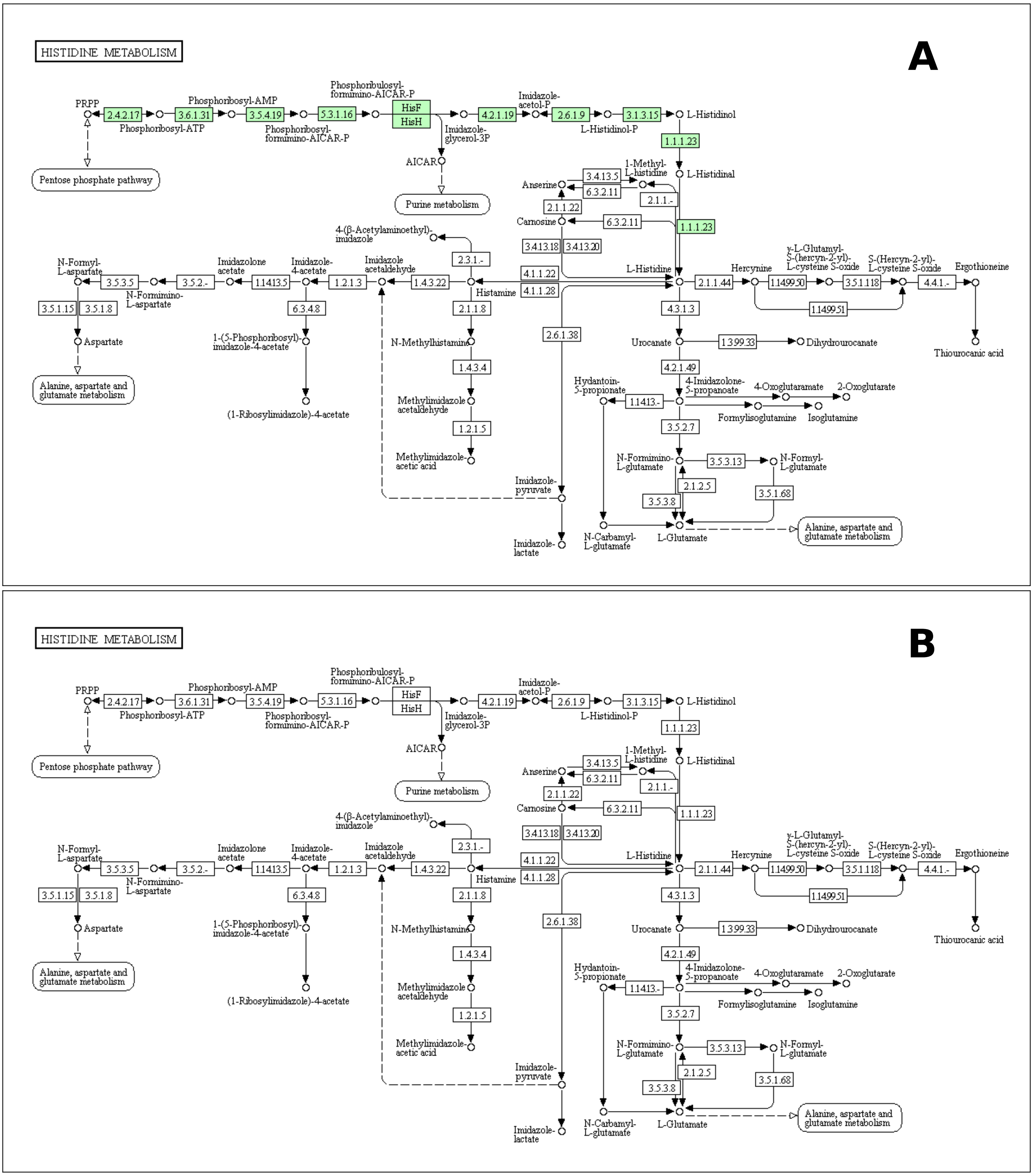

Figure S19. KEGG histidine metabolism pathway. **(A)**: the inoculated *L. plantarum* strain and the *L. brevis* bin both possess the indicated genes. **(B)**: *O. oeni* bins do not contain any of these genes.

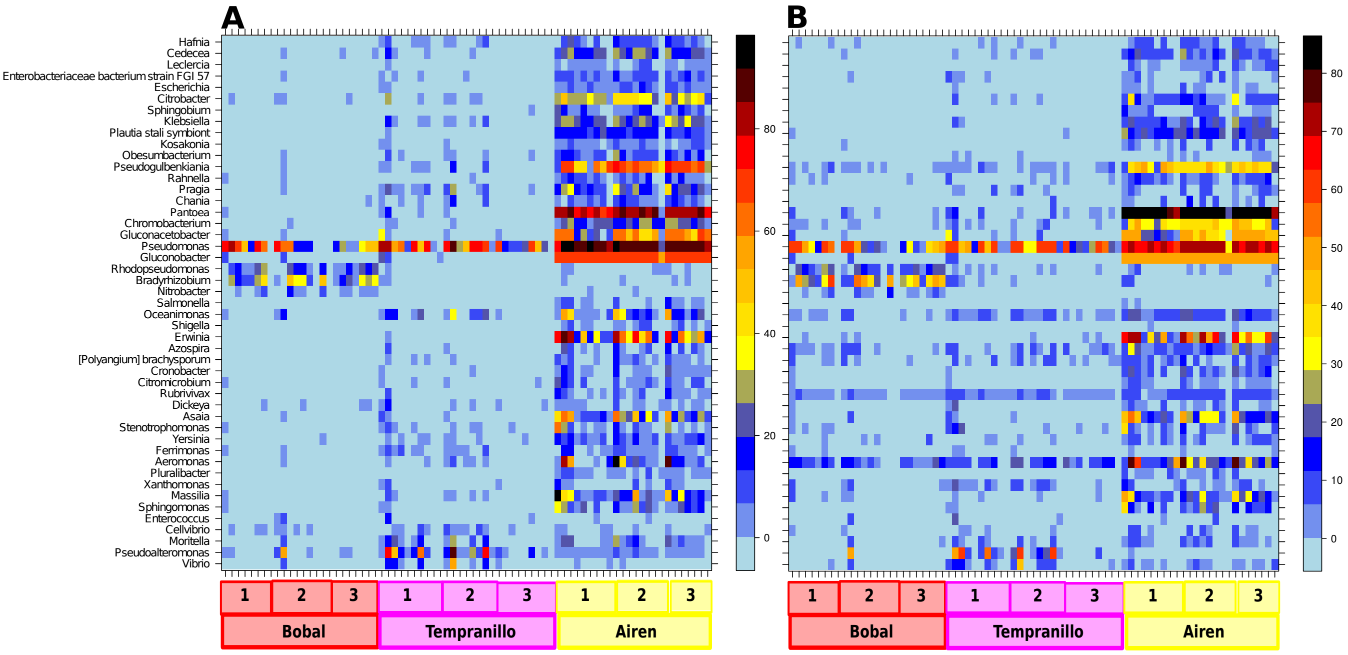

Figure S20. Heatmaps with genera of high pathway enrichment **(A)** on flagela and **(B)** on chemotaxis.

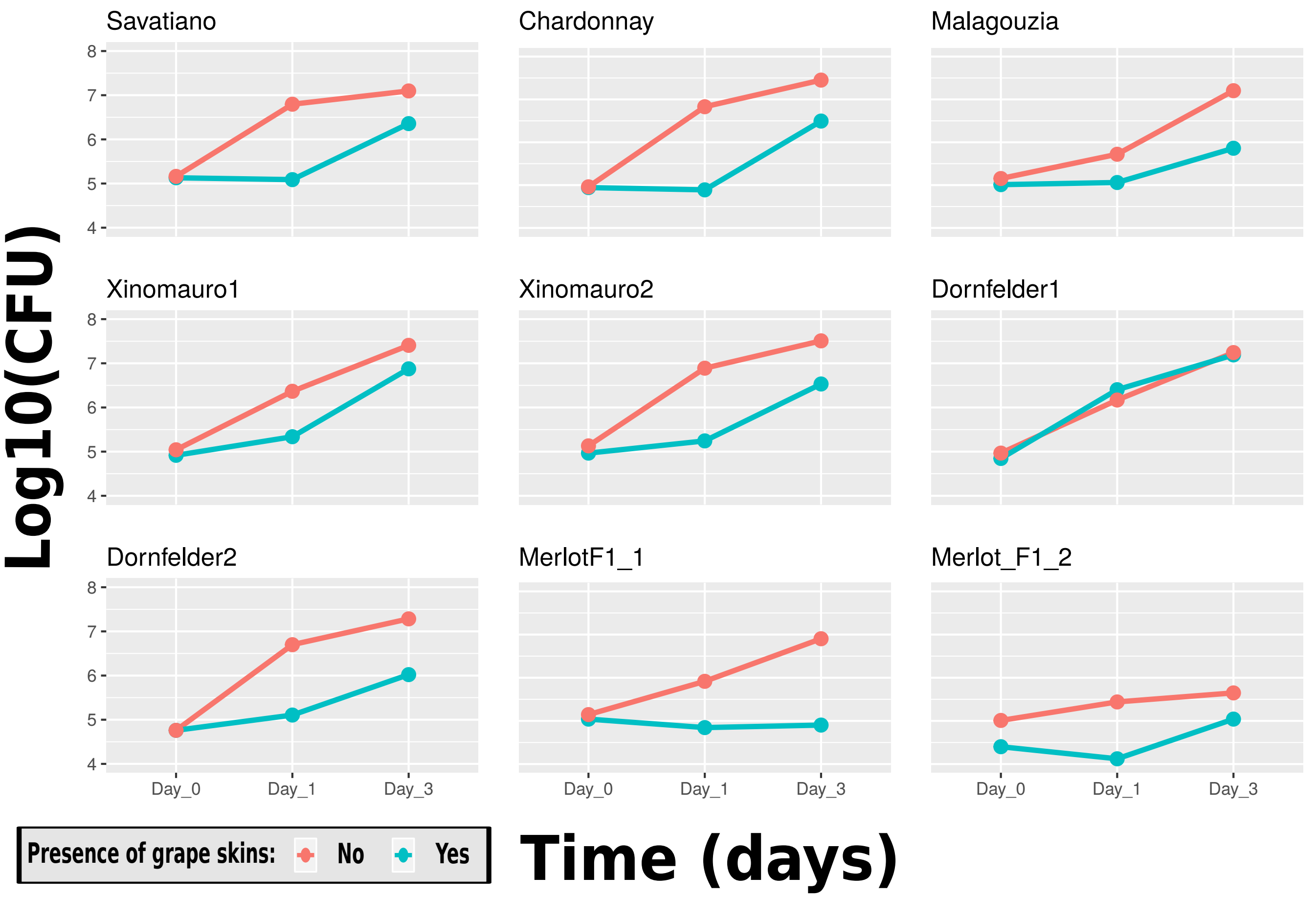

Figure S21. Nine different experiments on six different pasteurized grape juice varieties. *L. plantarum* was inoculated under two different conditions (presence of grape skins or without). The top row are white grape varieties while the rest are reds.

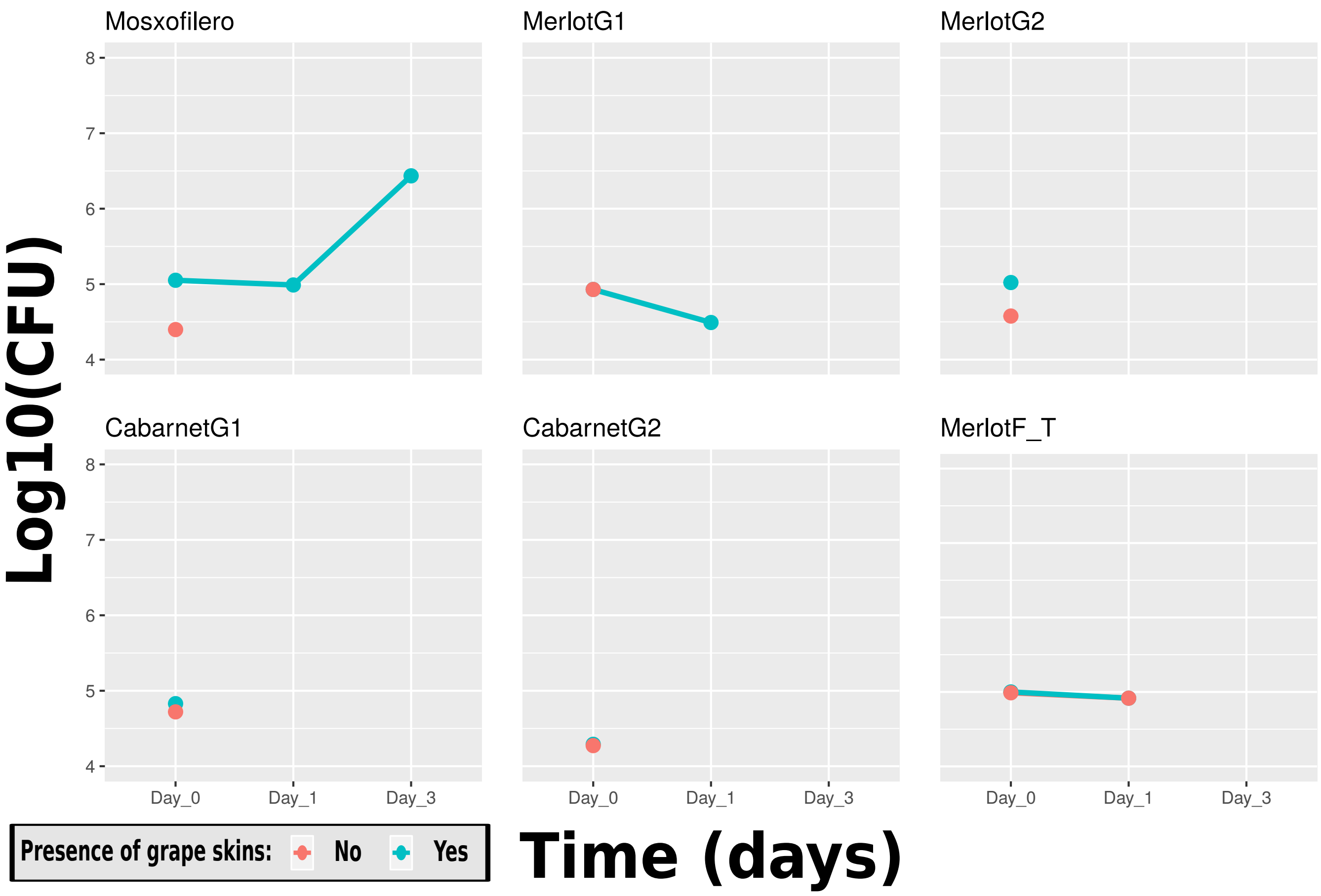

Figure S22. Six different experiments on four different pasteurized grape juice varieties. *L. plantarum* was inoculated under two different conditions (presence of grape skins or without), Mosxofilero is a white grape variety while the rest are red grapes. Absence of data points in the plots correspond to none or very small numbers of colonies.

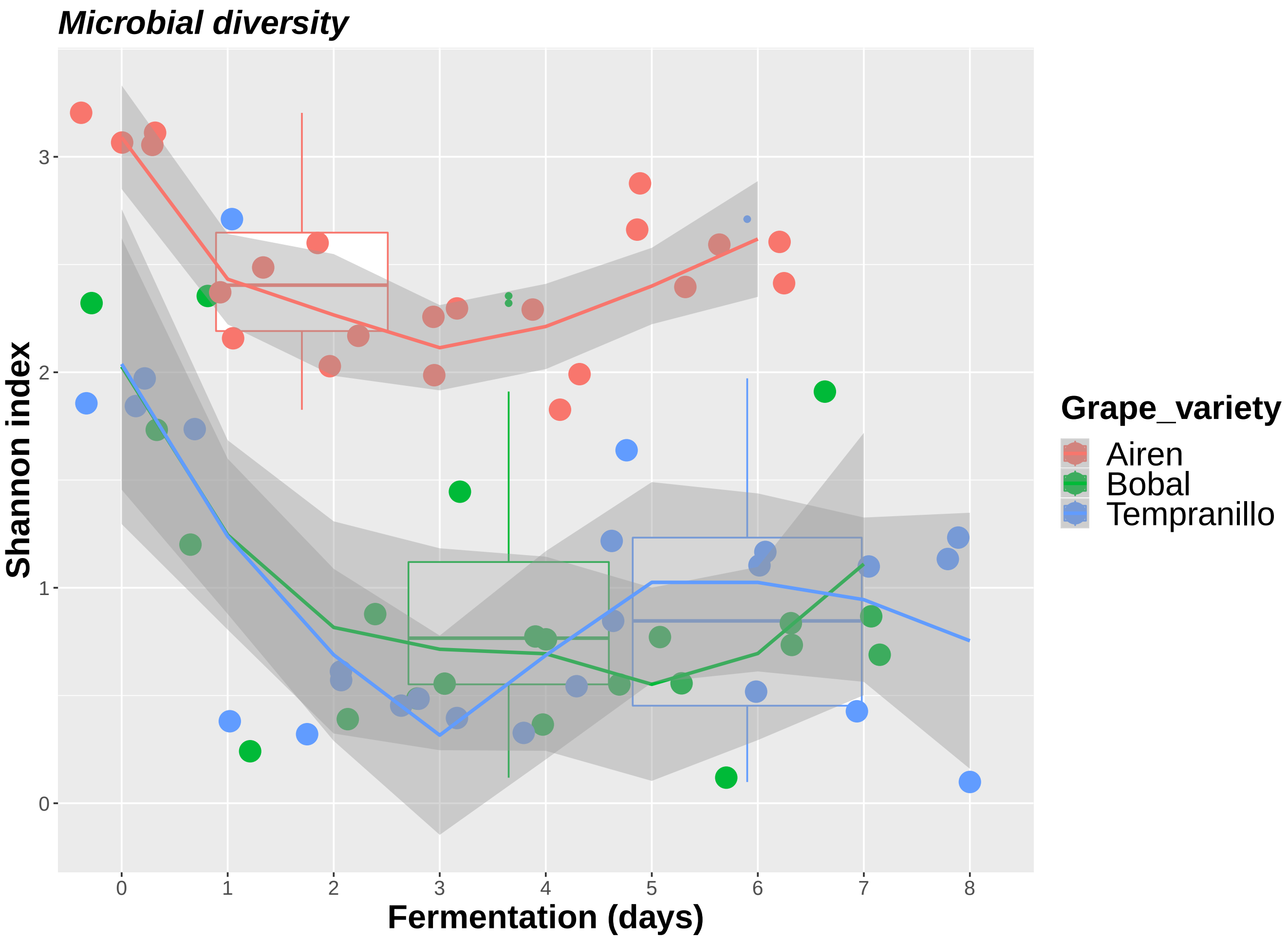

Figure S23. Shannon index of wine fermentations shows that Airen has the highest biodiversity compared to the two red wines (Bobal and Tempranillo).

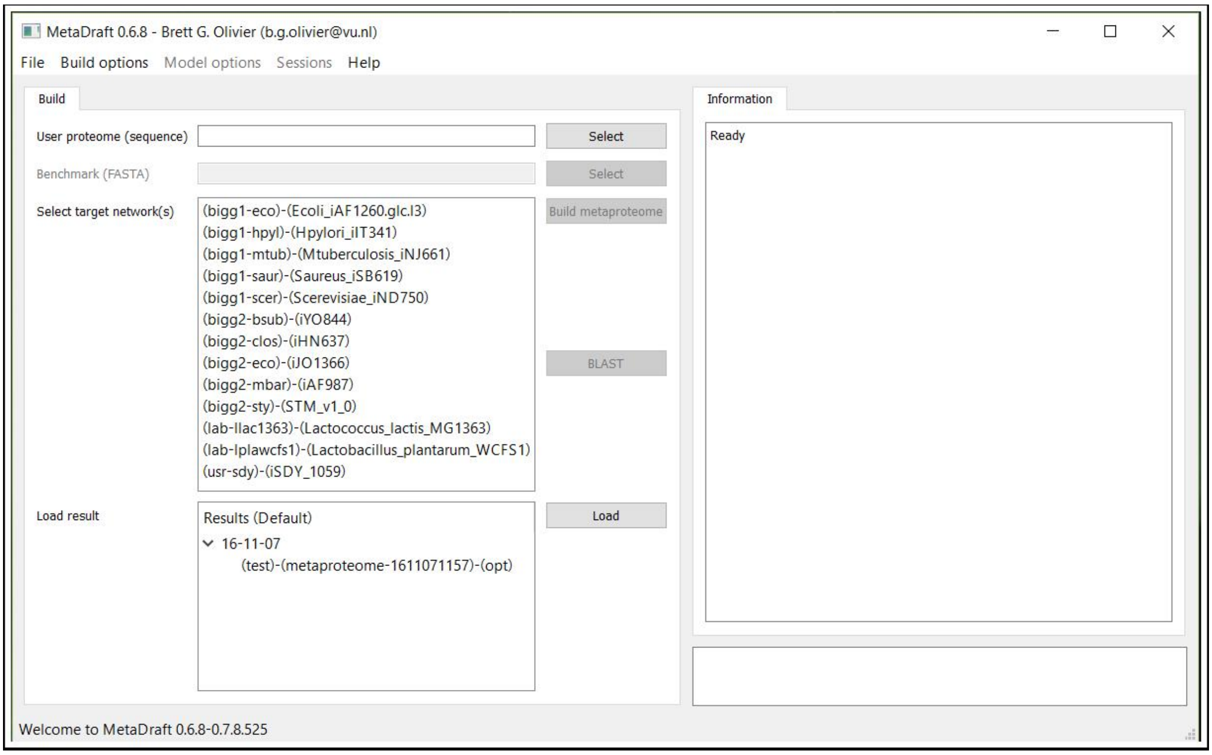

Figure S24. The Metadraft graphical user interface. The left-hand panel shows the template model selection list.

REFERENCES

Laczny, C. C., Pinel, N., Vlassis, N., and Wilmes, P. (2014). Alignment-free visualization of metagenomic data by nonlinear dimension reduction. *Scientific Reports* 4. doi:10.1038/srep04516

N, S., K, S., P, M., D, K., and N., P. (2011). The subliminal toolbox: automating steps in the reconstruction of metabolic networks. *J Integr Bioinform* doi:10.2390/biecoll-jib-2011-186

Notebaart, R. A., van Enckevort, F. H., Francke, C., Siezen, R. J., and Teusink, B. (2006). *BMC Bioinformatics* 7, 296. doi:10.1186/1471-2105-7-296

Overbeek, R., Begley, T., Butler, R. M., Choudhuri, J. V., Chuang, H.-Y., Cohoon, M., et al. (2005). The subsystems approach to genome annotation and its use in the project to annotate 1000 genomes. *Nucleic Acids Res* 33, 5691–5702. doi:10.1093/nar/gki866. 16214803[pmid]

Peng, Y., Leung, H. C. M., Yiu, S. M., and Chin, F. Y. L. (2012). Idba-ud: a de novo assembler for single-cell and metagenomic sequencing data with highly uneven depth. *Bioinformatics* 28, 1420–1428. doi:10.1093/bioinformatics/bts174

Thiele, I. and Palsson, B. Ø. (2010). A protocol for generating a high-quality genome-scale metabolic reconstruction. *Nature Protocols* 5, 93–121. doi:10.1038/nprot.2009.203

Wu, Y.-W., Simmons, B. A., and Singer, S. W. (2015). Maxbin 2.0: an automated binning algorithm to recover genomes from multiple metagenomic datasets. *Bioinformatics* 32, 605–607. doi:10.1093/bioinformatics/btv638

Wu, Y.-W., Tang, Y.-H., Tringe, S. G., Simmons, B. A., and Singer, S. W. (2014). Maxbin: an automated binning method to recover individual genomes from metagenomes using an expectation-maximization algorithm. *Microbiome* 2, 26. doi:10.1186/2049-2618-2-26
